# The involvement of oncobiosis and bacterial metabolite signaling in metastasis formation in breast cancer

**DOI:** 10.1007/s10555-021-10013-3

**Published:** 2021-12-30

**Authors:** Tünde Kovács, Edit Mikó, Gyula Ujlaki, Heba Yousef, Viktória Csontos, Karen Uray, Peter Bai

**Affiliations:** 1 Department Medical Chemistry, Faculty of Medicine, University of Debrecen, Debrecen, 4032 Hungary; 2 MTA-DE Lendület Laboratory of Cellular Metabolism, Debrecen, 4032 Hungary; 3 Research Center for Molecular Medicine, Faculty of Medicine, University of Debrecen, Debrecen, 4032 Hungary

**Keywords:** Breast cancer, Oncobiome, Oncobiosis, Inflammation, Metastasis, Bacterial metabolite

## Abstract

Breast cancer, the most frequent cancer in women, is characterized by pathological changes to the microbiome of breast tissue, the tumor, the gut, and the urinary tract. Changes to the microbiome are determined by the stage, grade, origin (NST/lobular), and receptor status of the tumor. This year is the 50th anniversary of when Hill and colleagues first showed that changes to the gut microbiome can support breast cancer growth, namely that the oncobiome can reactivate excreted estrogens. The currently available human and murine data suggest that oncobiosis is not a cause of breast cancer, but can support its growth. Furthermore, preexisting dysbiosis and the predisposition to cancer are transplantable. The breast’s and breast cancer’s inherent microbiome and the gut microbiome promote breast cancer growth by reactivating estrogens, rearranging cancer cell metabolism, bringing about a more inflammatory microenvironment, and reducing the number of tumor-infiltrating lymphocytes. Furthermore, the gut microbiome can produce cytostatic metabolites, the production of which decreases or blunts breast cancer. The role of oncobiosis in the urinary tract is largely uncharted. Oncobiosis in breast cancer supports invasion, metastasis, and recurrence by supporting cellular movement, epithelial-to-mesenchymal transition, cancer stem cell function, and diapedesis. Finally, the oncobiome can modify the pharmacokinetics of chemotherapeutic drugs. The microbiome provides novel leverage on breast cancer that should be exploited for better management of the disease.

## Introduction

Dysbiosis is an abnormal adaptation of the microbiome, characterized by abnormal microbial composition and function. Neoplastic diseases are characterized by dysbiosis that is coined oncobiosis [1]. The microbiome that is undergoing oncobiotic transformation is termed the oncobiome. Oncobiosis occurs in multiple neoplasias, including breast cancer, and oncobiosis may have a pathogenic role in these cancers [2–8]. In this review, we will dissect the microbiome-elicited pathways and discuss how these pathways protect against metastasis formation in breast cancer.

Breast cancer is the most frequent cancer among women and is the leading cause of cancer-related deaths in women [9, 10]. Nevertheless, in developed countries, the 5-year survival of breast cancer is above 80% due to population-wide screening programs and the consequent early identification [11]. Although several risk factors were identified that increase the risk for breast cancer, most newly diagnosed patients have no obvious risk factors [12]. The risk for breast cancer increases with age, and most breast cancer patients are diagnosed in their 50 s after menopause. Extended exposure to female hormones due to hormone-replacement therapy, early menarche, and late menopause are risk factors for breast cancer [12]. BRCA1 and BRCA2 genes were identified as genetic risk factors for breast cancer, although mutation carriers represent a minority among breast cancer patients [13]. A family history of breast cancer or neoplasias is also a risk factor for breast cancer [12] and dense breast [14, 15]. Successful pregnancies, lactation, and physical activity are protective factors against the disease [12, 16]. For further information, we refer the readers to the relevant guidelines [17–20]. We reference the latest versions of the guidelines. Nevertheless, we ask the readers to check whether the guidelines were updated at the time our paper is being read.

## Oncobiosis in breast cancer

The first mention of the possible pathological role of the oncobiome in breast cancer dates back to 1971 [21]. The causative role of oncobiosis in the pathogenesis of breast cancer is underscored by the observations that antibiotic use increases the risk for breast cancer in mice [22–24], and the majority of studies suggest an increased risk in humans, also [25–33] (it should be noted that [34] and [35] found no association between antibiotic exposure and breast cancer risk). In further support of the pathological role of the microbiome, prebiotics [36], probiotics [37–45], and diverse nutrition [46–49] reduce the risk of breast cancer. Furthermore, risk factors of breast cancer, such as high-density breast [50], early menarche [51], low physical activity [51], increases in BMI [51, 52], age [53], and alcohol consumption [54], are also associated with microbiome changes culminating in breast cancer–associated oncobiosis.

Multiple microbial compartments undergo oncobiotic transformation during breast cancer, including breast tissue [55, 56], milk ducts [57], the inherent microbiome of the breast carcinoma [54, 58–72], the distal gut [51, 52, 73–89], and the urinary microbiome [54, 90]. However, no differences in the microbiome of the nipple [57, 69] and the oral microbiome [54] between healthy individuals and breast cancer patients have been detected. Besides bacteria, viruses (parapoxviruses [91], human papillomavirus [92], Herpesviridae, Retroviridae, Parapoxviridae, Polyomaviridae, Papillomaviridae [64]), fungi, and parasites were identified in breast cancer tissue [64, 65, 70], although these signatures are not ubiquitous in all individuals. Of note, microbiome signatures in the oncobiome correlate with survival in breast cancer, which underlines the importance of oncobiotic transformation in regulating breast cancer behavior [70]. The microbiome is now considered a component of the tumor microenvironment [93].

**Fig. 1:**
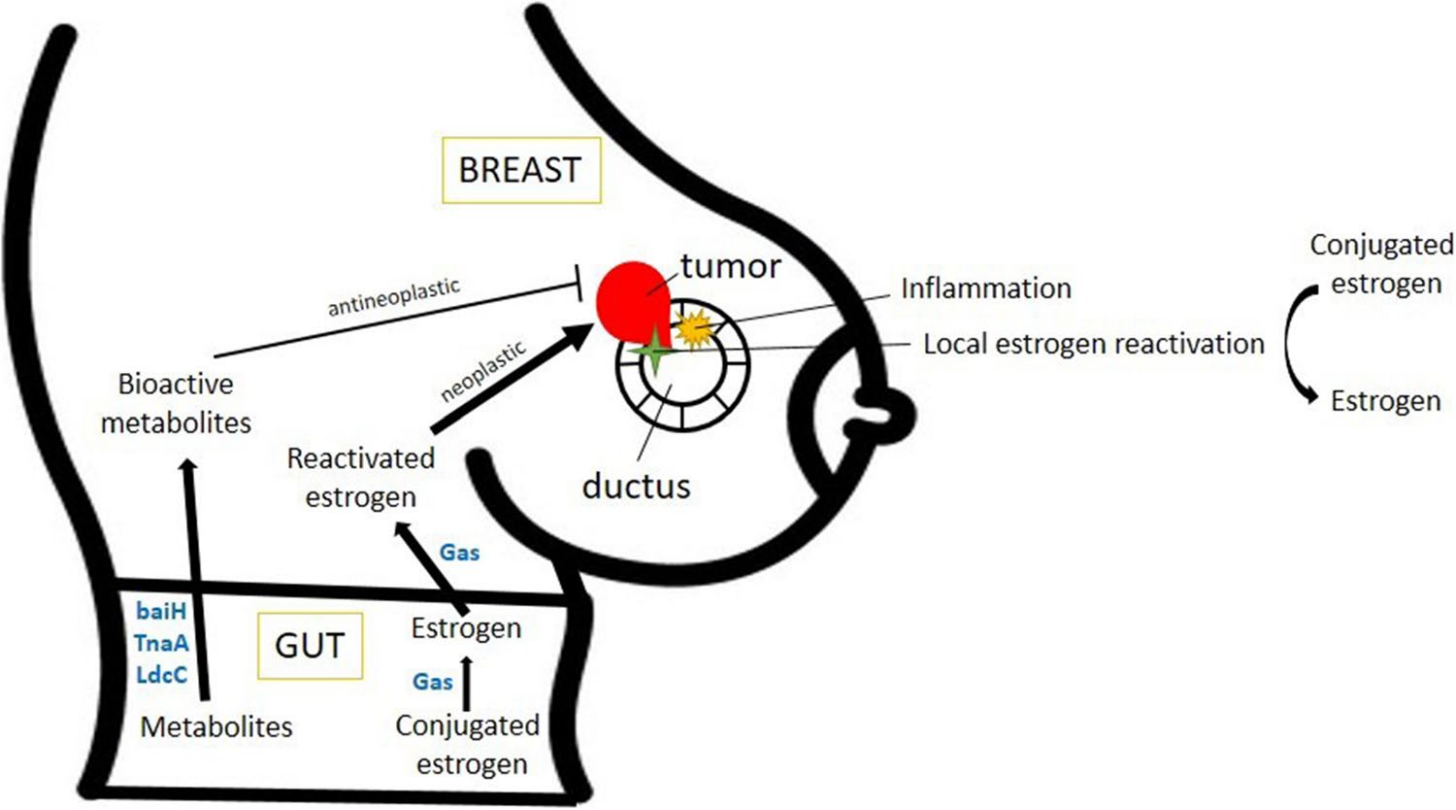
Oncobiosis supports breast carcinogenesis in a multi-pronged fashion. Abbreviations are in the text

In the following chapters, we will discuss changes in the microbiome of different compartments. In each compartment, the results can be contradictory, so we identified common elements that are discussed in the chapters discussing the respective compartments. The findings of the individual studies are assembled in Table 1. The bilateral mechanistic connections between the host and the microbiome [94] and oncobiosis support of cancer cells (Fig. 1) are discussed in the following chapters.

**Table 1:** Changes to the gut microbiome in breast cancer

Patient cohort Mode of analysis	Changes to the microbiome, biomarker observations, diversity	Reference
**Changes to the breast tissue microbiome**		
Breast tissue obtained from surgery of benign tumors (*n* = 13), cancerous (*n* = 45), and healthy breast tissue (*n* = 23) 16S rRNA gene sequencing	The bacterial composition of the healthy breast tissue and breast cancer tissue is different. Higher abundances of *Prevotella*, *Lactococcus*, *Streptococcus*, *Corynebacterium*, and *Micrococcus* in healthy breast tissue and *Bacillus*, *Staphylococcus*, unclassified *Enterobacteriaceae*, unclassified *Comamonadaceae*, and unclassified *Bacteroidetes* in breast cancer tissue	[55]
15 malignant cancer (stages I and II) and 13 benign atypia patients 16S rRNA gene sequencing	No significant differences in alpha diversity values, but beta diversity differs between the breast tissue of malignant and benign breast tissue. *Fusobacterium*, *Atopobium*, *Hydrogenophaga*, *Gluconacetobacter*, and *Lactobacillus* abundance increased in the tissue of the malignant cases	[56]
141 breast tissue samples from BC patients	Enterococcus abundance plays a vital role in regional recurrence	[95]
**Changes to the milk duct microbiome**		
Nipple aspirate fluid from breast cancer surviving patients (*n* = 6) and healthy controls (*n* = 9) 16S rRNA gene sequencing	Beta diversity, but not the alpha diversity, is different between breast cancer patients and healthy controls. *Alistipes* was present only in the nipple aspirate from breast cancer patients, while unclassified *Sphingomonadaceae* genus was enriched in the nipple aspirate of healthy controls	[57]
**Changes to the breast carcinoma microbiome**		
Percutan needle biopsy from 22 benign and 72 malignant breast cancer patients 16S rRNA gene sequencing	Slightly higher alpha diversity in patients with malignant disease. *Proteobacteria* increased in malignant cases. The genus *Propionicimonas* and the families *Micrococcaceae*, *Caulobacteraceae*, *Rhodobacteraceae*, *Nocardioidaceae*, and *Methylobacteriaceae* increased in the malignant disease group	[58]
8 normal breast tissues, 64 breast tumors, in 11 cases paired non-cancerous adjacent tissue 16S rRNA gene sequencing	Alpha diversity and beta diversity indices were lower in the tumor tissue. *Clostridia*, *Bacteroidia*, WPS_2, *Ruminococcaceae*, *Fusobacteria*, and *Spirochetes* increased, while *Pseudomonadaceae*, *Sphingomonadaceae*, *Caulobacteraceae*, *Thermi*, and *Actinobacteria* decreased in tumors. *Streptococcaceae* and *Ruminococcus* were abundant in TNBC tumors; *Xanthomonadales in* Luminal A; *Clostridium in* Luminal B*; Akkermansia. Ruminococcaceae* and genus *Hyphomicrobium* were more abundant in stage I Her2 + tumors, *Sporosarcina* in stage II, *Bosea* in stage III + IV	[59]
221 breast cancer specimens, breast tissue from 18 individuals predisposed to breast cancer, and 69 controls 16S rRNA gene sequencing	Alpha diversity values were lower in tumors and the breast tissue of the risk population. Widespread association with stage, lobular or ductal origin, and hormone receptor positivity	[60]
Cancerous tissue and adjacent healthy tissue from 16 breast cancer patients 16S rRNA gene sequencing	No significant differences in alpha diversity. No difference between the cancerous and the adjacent healthy tissues	[61]
BC tumor and adjacent normal tissue from 6 + 10 TNBC WNH and 7 TNBC BNH, 7 TPBC WNH, and 3 TPBC BNH 16S rRNA gene sequencing	In triple-positive and triple-negative breast cancer from black non-Hispanic alpha indices decrease, in white non-Hispanic women alpha indices increase *Fusobacteria* and *Streptococcus* abundance increase in the tumor. There is a difference between the microbiome composition of triple-positive and triple-negative breast cancers	[63]
10 archived breast cancer tumor tissue, 10 freshly excised normal breast tissue, 8 of them from both breasts 16S rRNA gene sequencing	*Ruminococcaceae*, *Akkermansia*, *Verrucomicrobia* increased, while *Sutterella*, *Acinetobacter Bacteroides*, *Cyanobacteria*, *Proteobacteria*, *Synergistetes*, and *Tenericutes* decreased in the tumor tissue. Alpha index decreased in the tumor tissue	[62]
44 BC patients and 20 controls. Significant age and body mass index difference between cohorts 16S rRNA gene sequencing	No significant difference in alpha diversity. *Methylobacterium* decreased in cancer patients and was drastically reduced when invasion was reported. In cancer patients, levels of gram-positive organisms including *Corynebacterium*, *Staphylococcus*, *Actinomyces*, and *Propionibacteriaceae* increased	[54]
100 TNBC, 17 matched, 20 non-matched controls PathoChip technology	Higher association of *Brevundimonas diminuta*, *Arcanobacterium haemolyticum*, *Peptoniphilus indolicus*, *Prevotella nigrescens*, *Propiniobacterium jensenii*, *and Capnocytophaga canimorsus* and a set of viruses and fungi are associated with TNBC samples compared to normal tissues	[64]
50 BRER, 34 BRHR, 24 BRTP, 40 TNBC, 20 controls PathoChip technology	BRER is characterized by *Arcanobacterium*, *Bifidobacterium*, *Cardiobacterium*, *Citrobacter*, and *Escherichia* species; BRTP is characterized by *Bordetella*, *Campylobacter*, *Chlamydia*, *Chlamydophila*, *Legionella*, and *Pasteurella*; BRHR is characterized by *Streptococcus;* TNBC is characterized by *Arcobacter*, *Geobacillus*, *Orientia*, and *Rothia*	[65]
Healthy (age-matched) (*n* = 23), paired normal (*n* = 39), and tumor tissue (*n* = 39) 16S rRNA gene sequencing	Bacterial copy number decreased in tumors and with increased grade. *Sphinomonadaceae* family, *Sphingomonas* species decreased, while the *Methylobacteriaceae* family, *Methylobacterium* species increased in tumors	[66]
668 breast tumor and 72 non-cancerous breast tumor sequences from the TCGA data portal	*Salmonella enterica*, *Escherichia coli*, *Bacillus alcalophilus*, *Brachybacterium muris*, *Plesonius fermentans*, *Mycobacterium phlei*, *and Acinetobacter radioresistens* increased, while *Microbacterium barkeri*, *Acinetobacterium baumannii*, *Ralstonia pickettii*, *Lactobacillus rossiae*, and *Mycobacterium fortuitum* decreased in the tumors	[68]
256 normal tissue and 355 breast tumors	Bacterial LPS and 16S RNA were detected in breast cancer cells in breast tumors. The microbiome of the breast tumors was richer than other tumors assessed and in normal adjacent breast tissue. *Proteobacteria*, *Firmicutes*, and *Actinobacteria* can be found in breast tumors. Differences in ER + and ER- breast tumors	[67]
21 female and 2 male BC patients 16S rRNA gene sequencing	Compared to normal breast tissue, the abundance of *Proteobacteria* increased in tumor tissue	[69]
95–105 FFPE samples for each BC subtype, 86 controls Pathochip	Large set of viruses, parasites, and fungi were detected in FFPE sections of breast cancer. The least of these were detected in TNBC cases	[70]
16 healthy controls, 32 breast cancer patients 16S rRNA gene sequencing	The abundance of Corynebacterium, Prevotella, and Gammaproteobacteria (unclassified) decreased, while Acinetobacter increased in BC tissue	[71]
**Changes to the gut microbiome**		
48 postmenopausal BC patients (most stages 0–I), vs. 48 control patients 16S rRNA gene sequencing	Breast cancer patients had a higher abundance of *Clostridium*, *Faecalibacterium*, and *Ruminococcus* (all *Clostridiales*) and a lower abundance of *Dorea* and *Lachnospiraceae* Lower number of observed species, Chao1 and PD whole tree indices in breast cancer patients	[73]
30 BC and 36 control patients Classical bacterial culture	Fecal bile acid levels were lower in breast cancer patients. Bacterial nuclear dehydrogenating activity increased in breast cancer patients suggesting increases in *Clostridia* abundance in feces	[82]
18 premenopausal BC patients, 25 premenopausal controls, 44 postmenopausal BC patients, 46 postmenopausal healthy controls Comprehensive shotgun sequencing	Species number, chao1, and JSD values were higher in postmenopausal cancer patients than in controls. Widespread taxonomical changes in BC patients	[75]
379 BC patients, 102 non-malignant breast disease, 414 population-based controls 16S rRNA gene sequencing	Alpha diversity indices correlate negatively with the odds for BC, BC grade, and subtype. No difference in the microbiome of malignant and non-malignant patients, but differ when compared to controls. Bacteroides, *Flavonifractor*, and *Ruminococcaceae* strongly and positively associated with BC, while *Rombutsia*, *Coprococcus* 2, *Christensenellaceae* R-7 group, *Dorea*, [Eubacterium] *coprostanoligenes* group, *Pseudobutyrivibrio*, and *Lachnospira* negatively associated with BC	[74]
32 overweight stage 0–II BC patients 16S rRNA gene sequencing	*Akkermansia* high abundance patients had higher alpha diversity compared to low abundance patients	[52]
31 female BC patients 16S rRNA gene sequencing	Total bacterial count decreased in overweight patients. The abundance of *Firmicutes*, *Faecalibacterium prausnitzii*, *Blautia* sp., and *Eggerthella lenta* decreased in overweight patients. *Blautia* sp. abundance increased as a function of tumor grade. *Bacteroidetes*, *Clostridium coccoides*, *Clostridium leptum*, *Faecalibacterium prausnitzii*, and *Blautia* sp. increased in stage II–III patients compared to stage 0–I patients	[77]
37 incident BC patients 16S rRNA gene sequencing	Early menarche patients had lower Chao1 and OTU indices and lower *Firmicutes* abundance. Lower OTU, Chao1, and Shannon indices; lower *Blautia*, *Coprococcus*, *Ruminococcus*, and *Oscillospira;* and higher *Firmicutes* abundance in Her2 − cases compared to Her2 + cases. *Clostridium* and *Vellionella* increased in grade III patients. High total body fat was associated with lower Chao1 and OTU indices	[51]
48 postmenopausal BC case patients (most stages 0–I), vs 48 control patients (same as [73]) qPCR of specific loci	Abundance of DNA coding for the baiH gene of *Clostridium sordelli*, *Bacteroides thetaiotaomicron*, *Escherichia coli*, *Pseudomonas putida*, *and Staphylococcus haemolyticus* decreased in breast cancer patients, the most pronounced changes were detected in in situ carcinoma patients	[78]
48 postmenopausal BC case patients (most stages 0–I), vs 48 control patients (same as [73]) qPCR of specific loci	Abundance of DNA coding for the CadA gene of *Escherichia coli* and the LdcC gene of *Escherichia coli*, *Enterobacter cloacae*, and *Hafnia alvei* decreased in breast cancer patients, the most pronounced changes were detected in in situ carcinoma patients	[79]
3 control and 4 stage I BC patients Western blotting	Fecal expression of *Escherichia coli* LdcC protein was lower in stage I patients	
48 postmenopausal BC case patients (most stages 0–I), vs 48 control patients (same as [73]) qPCR of specific loci	Abundance of DNA coding for the TnaA gene of *Alistipes shahii*, *Providencia rettegri*, *Bacteroides xylanisolvens*, and *Clostridium* sp. decreased in breast cancer patients, the most pronounced changes were detected in in situ carcinoma patients	[80]
35 BC patients Western blotting	Fecal expression of *Escherichia coli* TnaA was higher in patients with tumor-infiltrating lymphocyte (TIL) ratio over 20% compared to that of those below. Fecal *E. coli* TnaA expression correlated with TIL percentage	
35 BC cases Western blotting	Fecal expression of TnaA *Escherichia coli* LdcC protein was lower in lobular cases	[81]
48 postmenopausal BC cases, 48 control 16S rRNA gene sequencing	Lower alpha diversity in breast cancer patients. Lower alpha diversity among IgA-coated bacteria	[83]
124 BC survivor patients 16S rRNA gene sequencing	Abundance of *Actinobacteria* (*Bifidobacterium*) was associated with increased levels of DHA. Abundance of *Bacteroidetes* negatively correlated with EPA levels that were abrogated in patients receiving chemotherapy	[85]
30 controls vs. 25 BC cases 16S rRNA gene sequencing	In breast cancer patients, Bacteroidetes phylum, *Clostridium* cluster IV, *Clostridium* cluster XIVa, and *Blautia* sp. decreased, and Firmicutes phylum increased. No difference in the total number of bacteria. Alpha diversity increased in patients	[84]
200 BC patients (stages I–II) and 67 controls 16S rRNA gene sequencing	Alpha diversity lower in premenopausal patients, no difference in the postmenopausal cohort; beta diversity is different. Bacteroidetes proportions increased in BC patients	[87]
76 BC patients (35 stage II/III, 21 stage I), 336 healthy volunteers Comprehensive shotgun sequencing	52 units, mostly at the species level, decreased, while 38 units increased in breast cancer patients compared to healthy volunteers. 11 species increased in stage II/III patients, while 21 species increased in stage I patients	[86]
83 invasive BC patients, 19 patients with benign breast tumors 16S rRNA gene sequencing	No difference in alpha and beta diversity indices. The abundance of Clostridium, Faecalibacterium, Lachnospira, Erysipelotrichaceae, Romboutsia, Fusicatenibacter, Xylophilus, and Arcanobacterium decreased, while the abundance of Citrobacter increased in malignant BC patients. Distinct patterns identified BC subtypes and a microbial pattern associated with highly proliferative tumors	[89]
**Changes to the urinary microbiome**		
44 BC patients and 20 controls. Significant age and body mass index difference between cohorts 16S rRNA gene sequencing	Cancer patients had significantly higher Shannon index. Peri/postmenopausal urinary microbiome had higher Shannon index compared to premenopausal samples. *Corynebacterium*, *Staphylococcus*, *Actinomyces*, and Propionibacteriaceae abundance increased in cancer patients	[54]
220 controls and 127 BC patients 16S rRNA gene sequencing of the bacterial extracellular vesicles	The abundance of *Bacteroides* and *Ruminococcaceae*-derived extracellular vesicles were higher in the breast cancer group, while *Clostridiales* and *Pseudomonas*-derived extracellular vesicles were more abundant in healthy controls	[90]

Abbreviations: *BRHR*, human epidermal growth factor receptor 2 positive; *BMI*, body mass index; *BNH*, African American as Black non-Hispanic; *BRER*, estrogen or progesterone receptor positive; BRTP, estrogen, progesterone, and HER2 receptor positive; *EPA*, eicosapentaenoic acid; *FFPE*, formalin-fixed paraffin-embedded; *HER2*, epidermal growth factor receptor 2; *IDC*, invasive ductal carcinoma; *ILC*, invasive lobular carcinoma; *yrs*, years; *TIL*, tumor-infiltrating lymphocyte; *TNBC*, triple-negative breast cancer; *TPBC*, triple-positive breast cancer; *rRNA*, ribosomal RNA; *WNH*, White non-Hispanic

## Interactions between the oncobiome, tumors, and tumor cells

### Tumor colonization

The breast tissue has its own microbiome that has higher diversity than that of the vagina, but has lower diversity than that of the oral cavity or the gut [96]. The microbiomes of the breast skin and the inner breast tissues are different [56]. The microbiome of the exterior surface of the breast (e.g., the nipple) does not change in breast cancer [57]. Nevertheless, the composition of the microbiome of the milk ducts changes in the presence of a malignant process, evidenced in nipple aspirates [57].

Next-generation sequencing methods identified bacterial DNA in breast tumors [66] that was confirmed later by alternative methods [64, 65, 67]. Breast tumor has a higher bacterial load than melanoma, lung tumors, ovarian cancer, and glioblastoma and has similar counts to pancreas and bone cancers [67]. The carcinoma tissue is colonized by bacteria [54, 58–60, 62, 65, 67, 68, 70], and differences were detected among the subtypes of breast cancer as a function of hormone receptor status [54, 59, 60, 63–65, 70], HER2 receptor status [65], invasiveness [54], grade [54, 58, 60, 66, 70], stage [59, 60, 70], and immunological signatures [60]. Tumor-associated bacteria are culturable and class among *Proteobacteria*, *Firmicutes*, and *Actinobacteria* [67]. In a fraction of breast cancer cases, intracellular and perinuclear bacteria were identified [67]. Furthermore, fungi, parasites, and viruses [64, 70, 91, 92] were detected in tumor tissue. Racial differences were identified in the breast tissue and tumor microbiome [59, 63]. Of note, certain components of the intratumoral and tissular microbiome correlate with patient survival [70].

What are the functional contributions of the breast microbiome to tumorigenesis and tumor progression? As LPS + , Gram negative bacteria were detected intratumorally [67], intratumoral bacteria likely have a key role in the local immune response (see in a later chapter). In agreement with this concept, the expression of bacterial LPS biosynthetic genes were upregulated in nipple fluid aspirate [57]. Lipoteichoic acid (LTA), a marker for Gram positive bacteria, was absent in breast cancer [67].

Imputed pathway analysis revealed relevant functions for the breast microbiome (Table 2). As with other features, there are discrepancies between the studies. Nevertheless, the identified pathways can be classified into logical categories that can be linked to tumorigenesis. Furthermore, certain predicted metabolic pathways are similar to the pathway associated with carcinogenesis in breast tissue [62].

**Table 2:** Changes to the function of the oncobiome in breast cancer deducted from imputed pathway analyses

Bacterial pathway	Study
Stage I tumors were enriched in energy metabolism, fat digestion, and absorption	[59]
Stage II tumors are enriched in phosphotransferase system proteins	
Increased in benign cases:	[56]
Cysteine metabolism	
Methionine metabolism	
Glycosyltransferases	
Fatty acid biosynthesis	
Branched dibasic acid metabolism	
Increased in malignant cases:	
Drug metabolism (other enzymes)	
Inositol phosphate metabolism	
Upregulated in breast cancer cases:	[67]
Dermatan sulfate degradation	
Indole acetate biosynthesis	
L-ascorbate biosynthesis II (L-glucose pathway)	
Mycothiol biosynthesis	
Upregulated in breast cancer cases:	[55]
Colibactin biosynthesis	
Upregulated in breast cancer cases:	[57]
Flavone and flavonol biosynthesis (incl. beta-glucuronidase)	
Isoflavonoid biosynthesis	
Flavonoid biosynthesis	
Steroid hormone biosynthesis	
Synthesis and degradation of ketone bodies	
Tryptophan metabolism	
Sulfur metabolism	
Lipopolysaccharide biosynthesis	
Sphingolipid metabolism	
Polycyclic aromatic hydrocarbon degradation	
Glycine, serine, and threonine biosynthesis	
Oxidative phosphorylation	
Benzoate degradation	
Phenylalanine biosynthesis	
Peptidoglycan biosynthesis	
Linoleic acid biosynthesis	
Nitrogen metabolism	
Upregulated in breast cancer cases:	[62]
Base excision repair	
Th17 cell differentiation	
Choline metabolism	
Central carbon metabolism	
Necroptosis, microRNAs involved in carcinogenesis	
Proteoglycans involved in carcinogenesis	
Signaling pathways including IL-17, PI3K-Akt, HIF-1, and AMPK	

DNA coding for beta-glucuronidase enzymes (KEGG ortholog Beta-Glucuronidase K01195), responsible for conjugated estrogen reactivation, was elevated in the nipple aspirate fluid from breast cancer patients [57], suggesting a possible local reactivation of estrogens. In patients, *Roseburia*, *Rikenellaceae*, *Bacteroides uniformis*, *Paenibacillus amylolyticus*, and *Ellin6075* were the OTUs contributing to increased beta-glucuronidase abundance [57]. The same study showed an increase in the abundance of genes coding for steroid hormone biosynthesis [57].

Breast cancer cells are characterized by lower oxidative stress than the parent terminal lobular-ductal unit (TLDU) tissue [97]. The low level of oxidative and nitrosative stress is a key feature of successful tumor growth [98, 99]. Tissular and intratumor bacteria can support low tumoral oxidative and nitrosative stress by upregulating L-ascorbate biosynthesis II (L-glucose pathway) [67]. This is further strengthened by increased mycothiol biosynthesis in ER + tumors [67]. Mycothiol is used by bacteria to detoxify reactive oxygen species [100]. In addition to oxidative/nitrosative stress, the physical presence of bacteria can also induce DNA damage, at least in part, due to the overexpression of colibactin [55]. Among *Enterobacteriaceae*, *Escherichia coli* or *Staphylococcus* can produce a toxin, colibactin [55, 101, 102]. In agreement with this concept, Klann and colleagues [62] demonstrated a correlation between the expression of base excision repair genes and the bacterial colonization of the breast tissue.

Metabolic changes were described in the imputed pathway analyses. Of note, some reports show contradictory results. Changes affect core metabolic pathways, including anaerobic respiration [67], oxidative phosphorylation [57], and central carbon metabolism [62]; stage I tumors were enriched in genes of energy metabolism [59]. Another set of changes affected lipid metabolites, such as sphingolipid metabolism [57], synthesis and degradation of ketone bodies [57], linoleic acid biosynthesis [57], and choline metabolism [62]. In benign cases, fatty acid biosynthesis and branched dibasic acid metabolism increased [56], while in stage I tumors, genes of fat digestion and absorption were enriched [59]. A large set of amino acid metabolic pathways were affected (tryptophan glycine, serine, threonine, phenylalanine biosynthesis [57]), as well as nitrogen metabolism [57]. In benign cases, cysteine and methionine metabolism genes were enriched [56]. Changes were described in detoxification processes, such as polycyclic aromatic hydrocarbon degradation [57] and benzoate degradation [57] in the oncobiome. In malignant cases, local, oncobiome-mediated drug metabolism may increase [56]. Interestingly and importantly, signal transduction pathways such as PI3K-Akt [62], HIF-1 [62] and the AMPK pathway [62], microRNAs involved in carcinogenesis [62], and inositol phosphate metabolism [56] changed in the breast cancer oncobiome. In stage 2 tumors, the microbiome was enriched in phosphotransferase system protein genes [59]. Furthermore, sulfur metabolism genes changed in the breast cancer oncobiome [57] and glycosyltransferases increased in benign cases [56]. Breast cancer is characterized by changes in cell metabolism that is actionable for disease treatment and management [103–112]. Furthermore, the intricate supportive metabolic circuit between cancer cells and non-cancerous stroma cells can facilitate tumor growth and lead to worse clinical outcomes [103–105, 113]. Giallourou and colleagues [71] showed that breast bacteria interfere with the biosynthesis of ceramide, cholesterol, oxidized cholesteryl esters, diacylglycerol, lysophosphatidylcholine, phosphatidylethanolamines, and phosphatidylcholines to modulate the lipid composition of tumors. Of note, cholesterol and lipid homeostasis also play a role [4, 114–116]. The widespread changes to the oncobiome metabolism suggest that the breast cancer oncobiome participates in the metabolic support of rapidly dividing breast cancer cells.

In terms of cell death and cell division, the breast cancer oncobiome is associated with necroptosis [62]. *Haemophilus influenzae* is associated with mitotic spindle formation and G2M checkpoint regulation [68]. The breast cancer oncobiome is also associated with movement and metastasis-related processes, such as dermatan sulfate degradation [67], peptidoglycan biosynthesis [57], and proteoglycan homeostasis [62]. *Listeria fleischmannii* in the breast cancer oncobiome is associated with epithelial-to-mesenchymal transition [68].

Tzeng and colleagues have shown that elements of the microbiome covaried with different markers of bad clinical outcomes. Namely, *Staphylococcus* negatively covaried with *TRAF4*, *Pelomonas* positively covaried and *Bradyrhizobium* negatively covaried with VEGF-A, and *Propionibacterium* negatively covaried with PDGF-AA and PDGF-BB. In addition to these data, circumstantial data suggest bacterial secretory proteins [41] are probably also involved in communication between the microbiome and tumor tissue.

### Bacterial metabolite signaling and the oncobiosis of the gastrointestinal tract and urinary tract

The gut microbiome undergoes oncobiotic transformation in breast cancer [21, 51, 52, 73–82]. In terms of diversity indices, Goedert and colleagues [73] and Byrd and colleagues [74] reported lower or trends towards lower diversity indices in three different cohorts, while Zhu and colleagues [75] and Bobin-Dubigeon and co-workers [84] reported increased alpha diversity indices. Howe and co-workers [76] reported increases in alpha diversity indices in a Pten-deficient mouse model backing the observations of Zhu and colleagues [75] and Bobin-Dubigeon and co-workers [84]. No differences in alpha diversity were reported in [87] and [89]. However, the multiple observations make lower alpha diversity more likely. As noted earlier, risk factors for breast cancer lead to decreases in diversity, such as high-density breast [50], early menarche [51], low physical activity [51], and increases in BMI [51, 52]. Furthermore, antibiotic overdose, which leads to lower diversity, increases the risk for breast cancer [22, 23, 25–32], while probiotics that increase diversity have a protective effect [37–45].

Characteristic changes in the microbiome were observed between clinical stages [51, 77–79, 81] and grades [51, 89], MIB positivity [51], receptor status [51, 89], and proliferative capacity [89]. The most drastic changes were observed among in situ carcinoma and stage I patients, which were gradually rediversified in subsequent stages [77–79, 81]. Characteristic changes in taxa between patients and controls include *Clostridiales* [51, 73, 77, 82], *Blautia* [51, 77], and *Akkermansia muciniphila* [52, 76].

The gut microbiome is distant from the breast tumor; hence, signaling pathways are needed to connect the two distant compartments. Multiple pathways cross-connect the oncobiome and the tumor tissue. The direct immunomodulatory effects of the microbiome will be discussed in the next chapter.

Intestinal bacteria expressing beta galactosidases (gus and BC genes [117–119]) can deconjugate conjugated estrogens. The gus gene is widespread among bacteria, while changes in BC include *Bacteroidetes* and *Firmicutes* [119]. *Collinsella*, *Edwardsiella*, *Alistipes*, *Bacteroides*, *Bifidobacterium*, *Citrobacter*, *Clostridium*, *Dermabacter*, *Escherichia*, *Faecalibacterium*, *Lactobacillus*, *Marvinbryantia*, *Propionibacterium*, *Roseburia*, and *Tannerella* were shown to express β-glucuronidases [120]. Goedert and colleagues provided strong evidence for the involvement of *Clostridiales* in estrogen reactivation in breast cancer patients [73, 121, 122]. The oncobiome has increased capacity to reactivate estrogens [21, 50, 73, 74, 83, 121–124] enabling their reuptake and supporting the growth of estrogen-dependent, estrogen receptor–positive (ER +) breast tumors. Of note, the capacity for estrogen reactivation was identified by pathway analysis in the breast and nipple aspirate microbiomes [57].

Bioactive metabolites, synthesized by the microbiome or the oncobiome, can act in a similar fashion to hormones and can link up the microbiome and the distantly located cancer cells [2–4, 125]. As the gut microbiome is the biggest in the body in terms of the number of bacteria, its metabolic capacity is considerable. The biosynthetic capacity of the oncobiome is suppressed compared to the eubiome [126, 127]. Multiple bioactive bacterial metabolites were identified that can modulate the behavior of breast cancer cells (Table 3). The importance of changes to the metabolome in breast cancer is further highlighted by the large number of metabolomic studies that point towards the role of the metabolome in breast cancer incidence and evolution [128–132]. The bioactive bacterial metabolites are very chemically diverse. We will briefly highlight the most important bacterial metabolites that have cytostatic features. A set of bacterial toxins contributes to the oncogenic property of the oncobiome (Table 4) similar to other neoplasias [133–135].

**Table 3:** Bioactive microbial metabolites in breast cancer

Metabolite group	Made from	Producing bacteria		Relevant enzyme(s)		Receptor		Effect	
			Ref		Ref		Ref		Ref
Reactivated estrogens	Conjugated estrogens	*Firmicutes* *Collinsella* *Edwardsiella* *Alistipes* *Bacteroides* *Bifidobacterium* *Citrobacter* *Clostridium* *Dermabacter* *Escherichia* *Faecalibacterium* *Lactobacillus* *Marvinbryantia* *Propionibacterium* *Roseburia* *Tannerella*	[73, 117–119, 121, 122]	β-glucuronidase (gus/BC)	[117–119]	ERα ERβ mER (mERα, mERβ, GPER, GPRC6, ER-X, G_q_-mER)	[136, 137]	OXPHOS, tamoxifen resistance, metastasis, aggressivity, hormone-induced apoptosis, EMT, proliferation, metastasis	[138–146]
Short-chain fatty acids Acetate Butyrate Formate Lactate Propionate Pyruvate	Non-digestible carbohydrates, branched Chain amino acids	*Akkermansia muciniphila* *Lachnospiraceae* *Ruminococcus obeum* *Roseburia inulinivorans* *Bacteroidetes* *Negativicutes sp.* *Faecalibacterium* *Prausnitzii* *Eubacterium rectale* *Roseburia faecis* *Eubacterium hallii* *SS2/1* *Odoribacter* *Anaeotruncus*	[23, 147–149]	Thioesterases, phosphate acetyltransferase, acetate kinase, phosphate butyryltransferase, butyrate kinase, lactate dehydrogenase	[150]	FFAR HDAC AHR	[151–153]	OXPHOS (direct energy substrates), apoptosis, HDAC inhibition, macrophage antimicrobial activity	[154–158]
Secondary bile acids LCA DCA UDCA	CDCA CA 7-keto-litocholic acid	*Clostridium* *Enterococcus* *Bifidobacterium* *Lactobacillus* *Streptococcus* *Eubacterium* *Listeria* *Bacteroides* *Methanobrevibacter* *Methanosphera* *Escherichia* *Ruminococcus*	[159–166]	Bile salt hydrolases (BSH), 7α/β -hydroxysteroid, dehydroxylase (baiH)	[167]	TGR5 FXR SHP	[168–170]	Apoptosis, proliferation, VEGF production, OXPHOS, antitumor immunity, EMT, fatty acid biosynthesis, movement, metastasis formation, increased oxidative and nitrosative stress	[78, 97, 171]
Biologically active amines Cadaverine	L-lysine	*Shigella flexneri* *Shigella sonnei* *Escherichia coli* *Streptococci*	[172, 173]	Lysine decarboxylase (LdcC, CadA)	[172, 173]	TAAR1, 2, 3, 5, 8, 9	[174]	OXPHOS, CSC, movement, invasion, EMT, metastasis formation	[79]
Indole derivatives Indoxyl sulfate Indolepropionic acid	Tryptophan	*Providencia rettgeri* *Alistipes shahii* *Bacteroides xylanisolvens* *Clostridium* *Lactobacillus reuteri*	[81, 175, 176]	TnaA SULT1, Cyp2e1	[176] [176]	AHR PXR	[176] [81, 175]	OXPHOS, CSC, movement and proliferation, invasion, EMT, metastasis formation, antitumor immunity	[81, 175]

Abbreviations: *CA*, cholic acid; *CDCA*, chenodeoxycholic acid; *CSC*, cancer stem cell; *EMT*, epithelial-to-mesenchymal transition; *ER*, estrogen receptor; *FFAR*, free fatty acid receptor; *FXR*, farnesyl X receptor; *HDAC*, histone deacetylase; *LPA*, lysophosphatidic acid; *LPS*, lysophospholipids; *OXPHOS*, oxidative phosphorylation; *TAAR*, trace amine-related receptor; *TGR5/GPBAR1*, G protein-coupled bile acid receptor 1; *VEGF*, vascular endothelial growth factor

**Table 4:** Structural and secreted bacterial toxins supporting breast cancer

Metabolite group	Made from	Producing bacteria		Relevant enzyme(s)		Receptor		Effect	
			Ref		Ref		Ref		Ref
LPS	Lipid A + core oligosaccharide + O-specific polysaccharide	*Escherichia coli* *Salmonella enterica* *Vibrio cholera* *Pseudomonas* *Pantoea*	[177]	Lpx	[178, 179]	TLR2 TLR4	[177, 180]	Apoptosis, migration and metastases, EMT and β-catenin signaling, invasiveness	[181–183]
Lysophospholipids (LPS) Lysophosphatidic acid (LPA)	Phospholipid	*Vibrio cholerae* *Helicobacter pylori* *Yersinia pseudotuberculosis*	[184]	Phospholipase A2 Exogenous lipase	[184]	LPAR1-5	[185, 186]	Proliferation, migration, metastasis, stress fiber and focal adhesion formation	[187–189]
Colibactin	Precolibactin	*Escherichia coli* *Klebsiella pneumoniae* *Enterobacter aerogenes* *Citrobacter koseri*	[190]	ClbA-S	[190]	Unknown		Unknown	

Lithocholic acid (LCA) is a secondary bile acid derived from primary bile acids. Mostly *Clostridia* in the large intestines are responsible for the production of LCA and secondary bile acids in general [82]; however, other taxons are also involved [191]. The enzymes involved in secondary bile acid production are assembled in the bile acid inducible (bai) operon in bacteria [191]. The production of LCA from its precursors involves the deconjugation of primary bile acids and the oxidation, epimerization, and dehydroxylation of the gonane core [2, 4, 167, 191, 192]. Secondary bile acids, such as LCA, have pleiotropic roles in the microbiome, including regulation of the microbiome composition [193–200], facilitation of bacterial translocation into tissues [201], and quorum sensing [202, 203].

The bile acids in the breast are of the gut origin [204, 205]. Total bile acid levels in the serum of healthy individuals are lower than 5 µM. LCA serum reference concentrations are low (30–50 nM). However, tissue levels in the breast may be substantially higher [78, 206]. In breast cancer, both the hepatic synthesis of primary bile acids and the bacterial conversion to secondary bile acids in the large intestine are suppressed, and this suppression is the most dominant in in situ carcinoma and stage I patients [78, 82]. In good agreement with this concept, serum LCA levels negatively correlate with the Ki67 labeling index in breast cancer [207]. The composition of the serum bile acid pool in patients with benign breast disease is different from breast cancer patients; breast cancer patients had higher serum chenodeoxycholic acid levels and lower dihydroxytauro-conjugated bile acids (Tdi-1) and sulfated dihydroxyglyco-conjugated bile acids (Gdi-S-1) [208]. Another secondary bile acid deoxycholic acid (DCA) may act as a procarcinogenic agent [209, 210] and may be responsible for the procarcinogenic character of secondary bile acids [211].

A multitude of receptors is involved in bile acid signaling, including *Takeda* G protein-coupled receptor 5 (TGR5) and farnesoid X receptor (FXR), which are important for the current discussion. One study [212] suggested the use of bile acid-tamoxifen conjugates for breast cancer therapy.

Multiple amino acid catabolic products derived from lysine and tryptophan have cytostatic properties in breast cancer. Indole derivatives are made from tryptophan, while lysine decarboxylation yields cadaverine.

The microbiome accounts for 4–6% of tryptophan catabolism to yield indol derivatives [213], of which indolepropionic acid (IPA) and indoxyl sulfate (IS) have cytostatic properties in breast cancer [81, 175]. The serum reference concentration of IPA is submicromolar [176, 214, 215], while IS concentrations are low micromolar [216]. The bacterial enzyme responsible for IPA and IS biosynthesis, called tryptophanase (TnaA), can be found in the tryptophanase operon [176]. Tryptophanase expression is widespread among bacteria [217, 218]. IPA and IS, similar to other indole derivatives, can activate the aryl hydrocarbon receptor (AHR) and bind to the pregnane-X receptor (PXR) receptor [219–221]. Indole derivatives have a strong immunostimulatory effect [222–224]; IPA and IS can induce antitumor immunity in breast cancer [81, 175] and modulate the composition of the microbiome [219, 225–227]. Evidence from human studies supports the role of tryptophan and indole metabolism in breast cancer. Elevated extracellular tryptophan levels decrease survival in breast cancer (Table S8 [228]). Ki67 positivity negatively correlates with 3-indoxyl sulfate levels ([207] Additional file 9, Table S8 line 130), and 3-indoxyl sulfate levels are downregulated in both estrogen receptor–positive and negative cases ([207] Additional file 3, Table S3 line 44). TnaA DNA is downregulated in the breast cancer microbiome, and the most drastic changes were observed in in situ and stage I cases [81].

In the human body, cadaverine can be of bacterial, human, or nutritional origin. Nevertheless, bacterial cadaverine production seems to dominate [79]. In bacteria, the LdcC and CadA genes are responsible for cadaverine biosynthesis [172, 229], while diamino-oxidase eliminates cadaverine [230]. The capacity for cadaverine biosynthesis was identified in a number of bacteria [231–233]. Human serum reference concentration of cadaverine is submicromolar (100–800 nM) [234, 235]. Cadaverine can activate trace amine-associated receptors (TAAR1, 2, 3, 5, 8, 9), and these receptors are associated with breast cancer [79, 174]. Fecal TnaA protein content was reduced in E-cadherin-negative breast cancer cases compared to E-cadherin-positive cases [80].

Short-chain fatty acids (SCFAs), such as formate, acetate, propionate, butyrate, and lactate, are generated by a large set of bacterial species from non-digestible carbohydrates and a minor fraction from amino acids [236]. SCFAs are formed at multiple points in bacterial metabolism and are then released into the environment. Therefore, a wide circle of bacteria can synthesize SCFAs. The reference concentration of SCFAs in the human serum is in the range of 10–100 µM [237–239] and may reach up to 1 mM locally [240]. The receptors for SCFAs are the free fatty acid receptors (FFARs) and AHR [151].

Oncobiosis-related changes to bacterial metabolite production support oncogenesis and not tumor initiation through multi-faceted effects. In certain cases, as for SCFAs, effects are context-dependent; SCFAs can have positive (e.g., [241]) or negative (e.g., [242]) effects in breast cancer. Many studies used metabolites in superphysiological concentrations that may render the interpretation of these studies difficult [243–246]. In superphysiological concentrations, metabolites can induce cell death [171, 244–247] and other features (e.g., in superphysiological concentrations, LCA blocked fatty acid biosynthesis [171] or induced cell death and the expression of multidrug resistance proteins [247]) that is usually mediated by secondary receptors, such as FXR in the case of LCA [247]. The metabolites assessed in the context of breast cancer were ineffective on untransformed primary human fibroblasts at their reference concentration, suggesting selectivity towards transformed cells [78, 79, 81, 97, 175].

Metabolites elicit effects by reducing oxidative and nitrosative stress through induction of NRF2 expression and its downstream effector genes and suppressing inducible nitric oxide synthase (iNOS). These effects may also involve the catechol-quinone metabolites of estrone and estradiol [81, 97, 175, 248]. Furthermore, the oncobiome contributes to the induction of Warburg metabolism in tumor cells [78, 79, 81, 97, 175]. These basic processes support epithelial-to-mesenchymal transition [78, 79, 81, 249], migration and invasion [79, 81], increased aldehyde dehydrogenase-1 (ALDH1)–positive cancer stem cells [79, 81], and suppression of tumor immunity [22, 24, 78, 81, 175, 250, 251]. These processes together support metastasis formation [24, 78, 79, 250, 251] and disease recurrence [29]. In addition, most SCFAs can act as energy sources in cells [233] and may inhibit histone deacetylases to modulate epigenetics [152, 154–157, 241, 252–257]. LCA supplementation can reduce VEGF production by the implanted breast cancer cell in an animal model of breast cancer [78]. Furthermore, the oncobiome correlates with omega-3 polyunsaturated fatty acid homeostasis [85].

Our current understanding indicates that these metabolites only represent the tip of the iceberg. Multiple studies [75, 87, 89] identified imputed pathways that were differentially regulated in the oncobiome and eubiome in breast cancer patients (Table 5). In addition to the metabolic changes, a large set of transport systems are dysregulated in bacteria [75]. These findings highlight the widespread effects of bacterial bioactive metabolite production.

**Table 5:** Imputed metabolic pathways dysregulated in the gut oncobiome of breast cancer patients

Induced in BC patients	Decreased in BC patients	Ref
**Premenopausal patients** Beta oxidation Pyridoxal biosynthesis Pentose phosphate pathway (oxidative) Heparane sulfate degradation Entner-Duodoroff pathway	**Premenopausal patients** Uridine monophosphate biosynthesis Reductive pentose phosphate cycle (ribulose5P → glyceraldehyde3P) Pyruvate oxidation to acetyl-CoA Phosphatidylethanolamie biosynthesis Inosine monophosphate biosynthesis Glycolysis GABA biosynthesis Formaldehyde assimilation, serine pathway F-type ATPase Dicarboxylate pathway Pantothenate biosynthesis C5 isopernoid biosynthesis, non-mevalonate pathway C1-unit interconversion	[75]
**Postmenopausal patients** Ubiquinone biosynthesis Jasmonic acid biosynthesis Beta oxidation LPS biosynthesis Glyoxylate cycle		[75]
Meta cleavage pathway of aromatic compounds Aromatic biogenic amine degradation Androstenedione degradation		[87]
LPS biosynthesis Ubiquinone and other terpenoid-quinone biosynthesis Folate biosynthesis Aminobenzoate degradation Biotin metabolism Glutathione metabolism Penicillin and cephalosporin biosynthesis D-Arginine and D-ornithine metabolism N-glycan biosynthesis Isoquinoline alkaloid biosynthesis Styrene degradation TCA cycle Geraniol degradation Indole alkaloid biosynthesis	Glycolysis/gluconeogenesis Glycerophospholipid metabolism	[89]

*BC*, breast cancer; *CoA*, coenzyme A; *GABA*, gamma-aminobutyric acid; *LPS*, lipopolysaccharide; *TCA*, tricarboxylic acid cycle

**Fig. 2:**
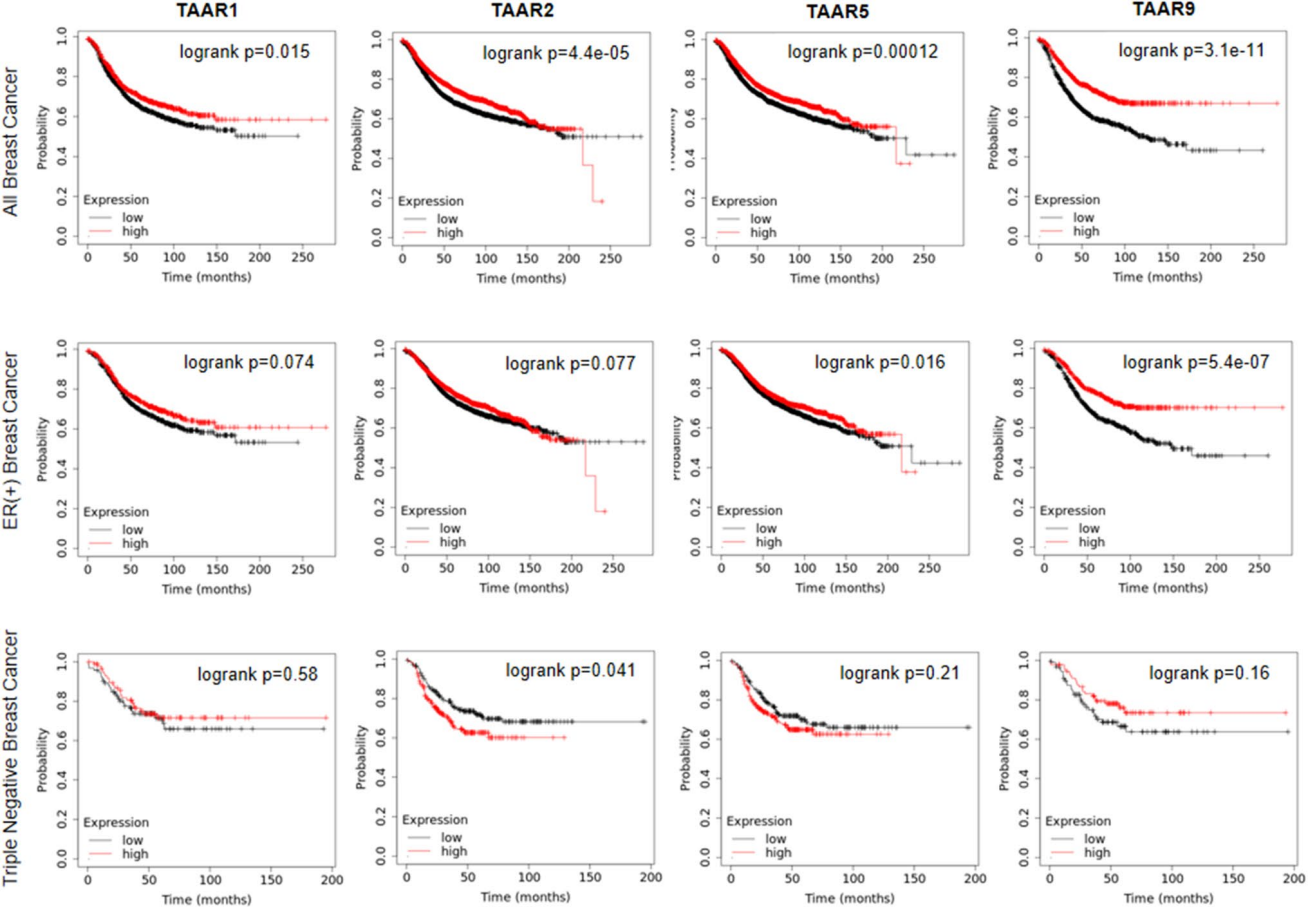
High expression of a subset of TAAR receptors prolongs relapse-free survival in breast cancer patients that is abrogated in TNBC cases. Survival curves were obtained from the kmplot.com site [258] on the 7th of October 2021

**Fig. 3:**
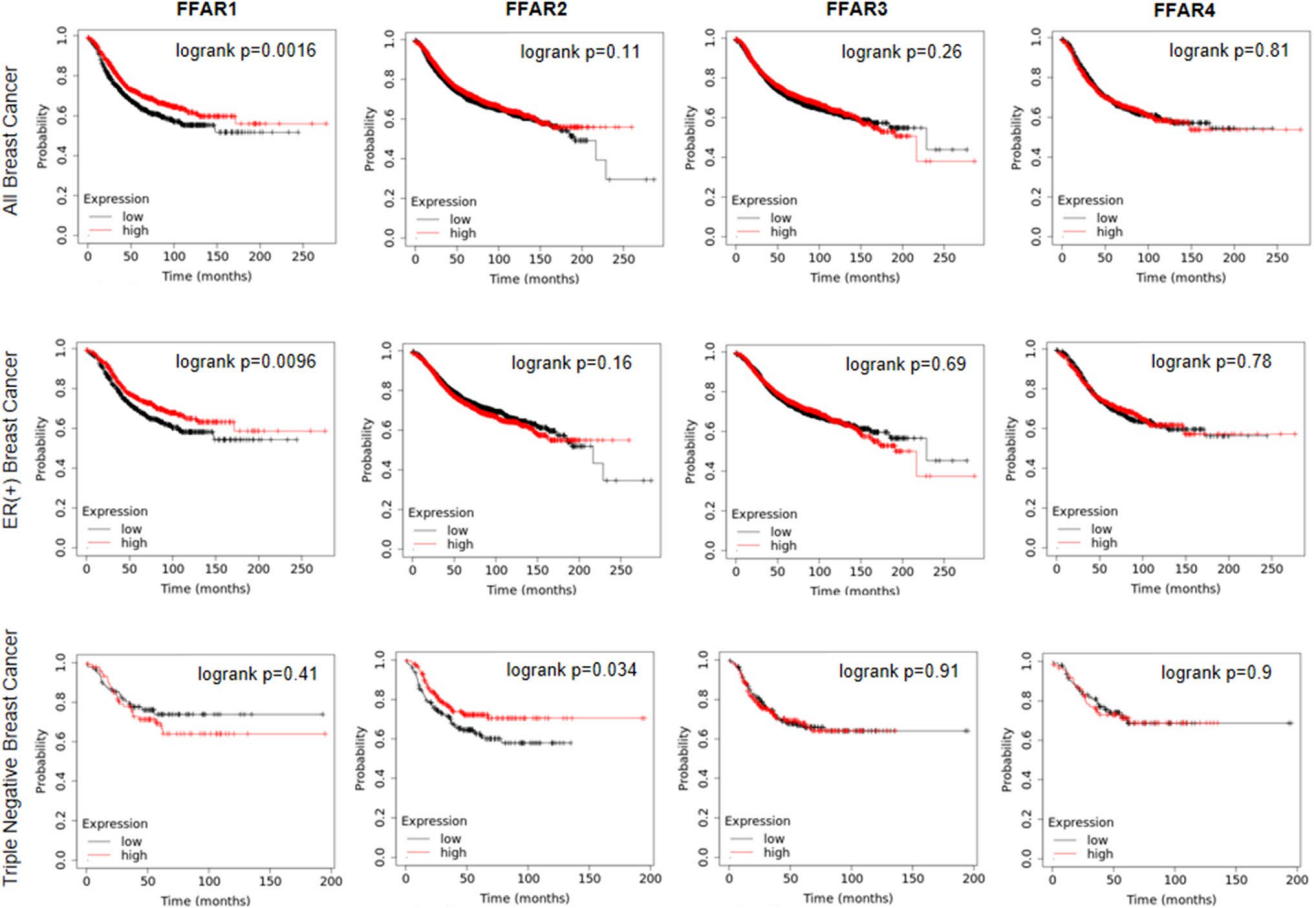
High expression of FFAR1 receptors prolongs relapse-free survival in breast cancer patients that is abrogated in TNBC cases. Survival curves were obtained from the kmplot.com site [258] on the 7th of October 2021

As mentioned earlier, the suppression of the biosynthetic capacity of the microbiome is most pronounced in the early stages of the disease [79, 81, 97]. Nevertheless, studies on the expression of receptors for the metabolites suggest that receptors on the surface of tumor cells are downregulated as tumor stage or grade increases [81, 97]. The receptors for microbiome-derived metabolites and the components of the downstream signaling pathways in the tumor correlate with the receptor status, grade, and stage. The intratumoral expression of AHR, PXR, and TGR5 decreases in TNBC cases compared to ER + cases [81, 97]. These findings were mirrored in relapse-free survival rates, where high expression of the receptors and their downstream signaling components provided better survival for patients that was abrogated in TNBC cases [81, 97]. In Figs. 2 and 3, we show that a subset of TAAR receptors and FFAR1 receptors have similar properties. Similarly, in LCA-elicited downstream signaling events, PXR and AHR receptor expression decreased as a function of stage, grade, or high mitotic rate [81, 97].

The impact of changes in the urinary microbiome is unexplored. However, recently, An and colleagues showed that bacterial extracellular vesicles can be isolated from the urine of healthy subjects and breast cancer patients and these extracellular vesicles can affect breast cancer cells differently [90].

### Antitumor immune responses

In general, the diverse status of the microbiome supports normal immune responses that are crucial for antitumor immunity [259–261]. Both the breast and the gut oncobiome have altered interactions with the immune system that we will review here. Whether changes to the bacterial community lead to changes to the immune system or the immune system causes changes to the microbiome remains an open question. Furthermore, myeloid and lymphoid infiltrations in tumors have differential effects; lymphoid infiltration is generally considered to have antitumor effects, while neutrophils are considered proneoplastic [262, 263]. We will discuss the microbiome-mediated changes to the immune system separately for the breast microbiome and the gut microbiome.

Before going into details, we would like to briefly introduce two bacterial immunogenic toxins, which are classified as pathogen-associated molecular patterns (PAMP). First, lipopolysaccharides (LPS) (Table 4), lypoglycans, or endotoxins are constituents of the outer membrane of Gram negative bacteria [177]. Toll-like receptor 2 and 4 respond to LPS stimulation [177, 180]. Second, lysophosphatids (Table 4) are generated in reactions related to bacterial membrane homeostasis [184, 264]. Cells of the host organism can also generate lysophosphatids. Gram negative bacteria tend to generate lysophosphatids [184, 264]. Endogenous phospholipase A2 or other exogenous lipases can generate lysophosphatids [184]. Lysophosphatids exert their effects through lysophosphatidic acid receptors (LPAR1-6) [185].

#### The breast’s inherent microbiome and immune responses

The inherent microbiome of the breast and the tumor is enriched in Gram negative bacteria [67], while lipotheichoic acid, specific for Gram positive bacteria, is absent in tumors [67]. These culturable bacteria are from *Proteobacteria*, *Firmicutes*, and *Actinobacteria* [67]. Alongside these findings, LPS was detected in tumor samples [67]. Bacterial LPS and 16*S* rRNA were demonstrated in CD45 + /CD68 − cells of a highly inflamed breast tumor, indicating that the colonizing bacteria tune and activate the immune system [67]. In the nipple fluid aspirate of breast cancer patients, the genes for LPS and lysophosphatid biosynthesis were upregulated in an imputed pathway analysis [57]. Similarly, imputed pathway analysis genes related to Th17 cell differentiation were enriched in the breast microbiome of breast cancer patients [62]. Further underlining these observations, bacterial peptidoglycan rendered breast cancer cells more invasive through activation of TLR2 receptors [261]. High intratumoral (antibacterial) inflammation can be boosted or sustained by viral infection [64, 91, 92]. In fact, human papillomavirus infection induces Stat3-activation and IL-17 expression in breast cancer patients [92]. Van der Merwe and colleagues [265] identified *Fusobacterium nucleatum* as a major species in the breast microbiome that overgrows in breast cancer patients, exerts an immunosuppressive phenotype, and activates the TLR4 receptor leading to immunosuppression.

Nevertheless, two studies [60, 66] refuted these observations. These studies found an association between microbiome components and inflammatory signaling. Namely, *Methylibium* and *Enhydrobacter* positively covaried with TLR signaling (*TLR3*, *TLR4*, *IRAK1*) in healthy control networks [60] and *Streptococcus* positively associated with *CD6*, *LAG3*, *SH2D1A*, and *TIGIT* expression and with T cell abundance in healthy control tissue [66]. However, in the tumor tissue, these associations [60] were lost, and the expression of antibacterial response genes (TLR2, TLR5, TLR9, NOD1, NOD2, CARD6, CARD9, TRAF6, borderline significant NFκB, BPI, IL-12A, MPO, and PRNT3) was lost [66]. Furthermore, in tumor tissue, *Methylibium* showed significant negative correlations with *ICOS* and *TBX21* expression and with T cell abundance [66]. These data [60, 66] contradict the data presented by other studies [57, 62, 67, 92, 261], and this discrepancy has not been explained yet.

#### The gut microbiome and immune responses

Immune responses likely play a role in the oncobiotic transformation of the gut microbiome, as multiple species, such as *Ruminococcus and Alistipes*, were opsonized in the stool of breast cancer patients [83]. On the other hand, the gut microbiome plays a pivotal role in fine-tuning the host’s immune system. In a large-cohort fecal microbiome study, multiple taxa immune-related functions were strongly associated with breast cancer [74]. In murine models, *Lactobacillus acidophilus* [39] or *Helicobacter hepaticus* [266] were associated with immune function in breast cancer. Oral gavage of *Helicobacter hepaticus* promotes mammary tumorigenesis that is dependent on the recruitment of neutrophils to the tumor; depletion of neutrophils using a monoclonal antibody abolished the promoter effect of *Helicobacter hepaticus* [266]. Oral treatment of mice with *Lactobacillus acidophilus* improved the immune response against the experimental tumor, reduced tumor growth, and tuned the immune response towards a Th1-type response [39]. Dysbiosis in the gut is associated with enhanced macrophage infiltration (CD11b + cells) to the breast tissue, of which the majority were M2-polarized (tolerogenic) macrophages [24]. Dysbiosis enhanced the mammary content of GM-CSF, IL23, IL22, CXCL1, and CCL2 in pretumor breast tissue that was further exacerbated in mice bearing breast tumors [24]. Mast cells also play a role in the oncobiome-induced immune effects [22].

Multiple gut-derived bacterial metabolites possess immunomodulatory roles in breast tumors. Lithocholic acid, indolepropionic acid, and indoxyl sulfate are produced in the intestines and can regulate tumor immune response against cancer cells in experimental models of breast cancer [78, 81, 175]. Butyrate may have immunomodulatory roles at multiple levels that were not directly assessed in breast cancer [267].

## The role of oncobiosis in metastasis formation, survival, and recurrence in breast cancer

Reports unanimously show that dysbiosis supports invasion and metastasis formation [24, 54, 78, 79, 250, 251]. However, reports concerning the association of species with node positivity or metastasis formation are divergent. In the breast microbiome, *Methylobacterium* decreased in cases with lymphovascular invasion [54]. In another study [60], node positivity was associated with *Acinetobacter* and *Bacteroides* and negatively associated with *Achromobacter.* Lymphovascular invasion was positively associated with *Lactobacillus* and negatively associated with *Alkanindiges* abundances. Reduced *Oblitimonas* abundance was associated with both lymphovascular invasion and node-positive status. *Brevundimonas* abundance increased in patients developing distant metastases [250].

**Fig. 4:**
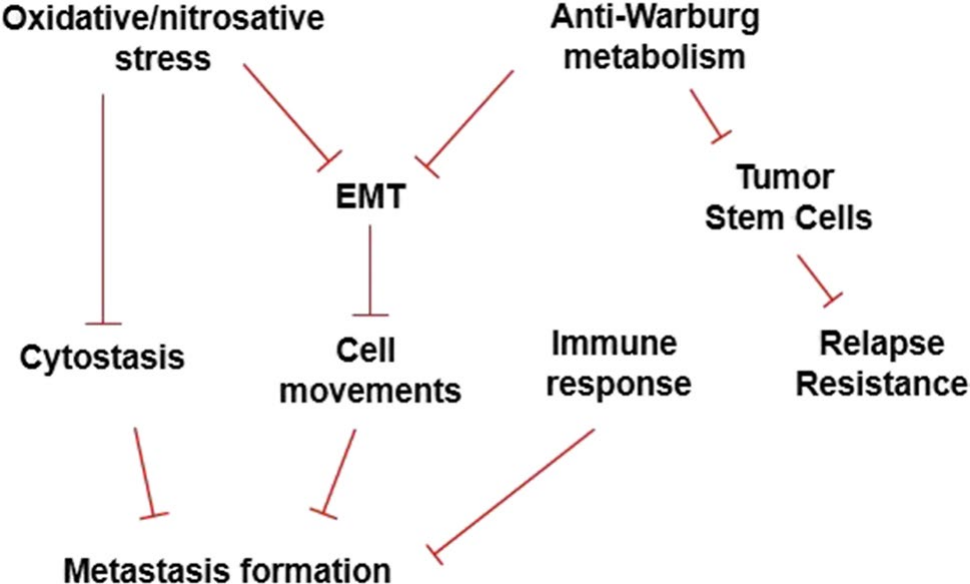
An overview of the processes through which the healthy eubiome suppresses metastasis formation and supports recurrence in breast cancer. These processes are lost in breast cancer–associated oncobiosis

What are the modalities through which oncobiosis can support metastasis formation? The oncobiome-elicited anti-metastatic and anti-recurrence effects on breast cancer are multi-pronged (Fig. 4). The breast cancer microbiome is associated with extracellular matrix degradation (e.g., dermatan sulfate degradation [67], suppression of peptidoglycan biosynthesis [57], and proteoglycans homeostasis [62]) that can support cellular movement within the tissue. The physical presence of bacteria can promote breast cancer cell invasiveness by activating toll-like receptor 2 on cancer cells via bacterial peptidoglycan [261]. The oncobiosis of the GI tract can also support cellular movement. For example, cadaverine, a cytostatic bacterial metabolite of the GI tract, can suppress breast cancer cell movement in in vitro assays and suppress matrix metalloproteinase-9 expression [79]. Furthermore, fecal TnaA protein content, responsible for bacterial cadaverine biosynthesis, is reduced in E-cadherin negative breast cancer cases compared to E-cadherin positive cases [80].

Improved tissular displacement of cancer cells accompanies the process of epithelial-to-mesenchymal transition (EMT). Intratumoral *Listeria fleischmannii* is associated with EMT [68]. Cytostatic bacterial metabolites (lithocholic acid, cadaverine, indoxyl sulfate, indolepropionic acid), which are lost in breast cancer, can suppress EMT [78, 79, 81, 175]. These metabolites can improve the expression of E-cadherin and ZO-1 and mesenchymal markers (Vim, Fgfbp1, Tgfb3, MMP9, SnaiI, β-catenin) [78, 79, 81, 249]. Altogether, these processes support migration and invasion [79, 81].

A very important contribution from Buchta Rosean [24] provided evidence that preexisting dysbiosis of the GI tract, induced by antibiotic treatment, supports tumor growth and metastasis formation in a murine model of breast cancer. Gut dysbiosis/oncobiosis induces an inflammatory response in the breast tumor, meloid cell infiltration to tumors, tumor fibrosis, and tumor dissemination. These proneoplastic, prometastatic traits can be induced in mice by transfer of fecal content from tumor-bearing mice.

In addition to cellular movement and EMT regulation, bacterial metabolites from the GI tract, such as lithocholic acid [78, 97] cadaverine [79], indolepropionic acid [81], and indoxyl sulfate [175], can suppress cellular proliferation. Furthermore, lithocholic acid reduces VEGF expression in experimental tumors [78]. These processes decrease the likelihood of tumor growth and blood or lymph vessel infiltration. The synthesis of these bioactive metabolites is suppressed in breast cancer patients [78, 79, 81]. An interesting observation from Absil and colleagues suggests that FXR, a bile acid receptor, plays a major role in setting the osteotropism of breast cancer cells [268].

The microbiome apparently has an impact on the neutrophil-to-lymphocyte ratio, which is a prognostic factor in breast cancer patients [263, 269]. Higher proportions of lymphocytes support antitumor immunity and reduce metastatic behavior, while higher neutrophil ratios support cancer progression and metastasis formation. As noted above, orally administered *Helicobacter hepaticus* promotes mammary tumorigenesis through neutrophil recruitment to tumors [266]. In contrast, among the bacterial metabolites, lithocholic acid [78] and indolepropionic acid [81] can induce higher proportions of tumor-infiltrating lymphocytes.

The composition of the oncobiome changes as a function of disease stage [60, 78, 79, 81] in breast cancer and, hence, is associated with survival. Antibiotic overdosing, which induces oncobiosis and supports the development of breast cancer, can increase the frequency of disease recurrence [29]. Components of the intratumoral and tissular microbiome correlate with patient survival [70, 72]. Based on the assessment of over 1000 archived breast cancer tissue samples, 46 bacteria were identified as risk factors (including *Leptospira*, *Desulfotalea*, *Archangium*, *Dicipivirus*, *Halosimplex*, *Spo1virus*, *Candidatus_Amoebophilus*, *Roseibium*, and *Arcticibacter*), while 48 bacteria were identified as favorable factors (including *Gordonia*, *Planktothricoides*, *Lachnoclostridium*, *Bafinivirus*, *Actinomadura*, and *Methanothermus*) [72]. Mao and colleagues [72] identified bacterial patterns in breast cancer tissue that strongly correlate with prognosis; poor and good prognosis patients could be separated based on these bacterial patterns (AUC > 0.8). Terrisse et al. [86] demonstrated that stool/fecal transfer of the human microbiome into tumor-bearing mice transformed the properties of the mouse tumors to the human counterpart (e.g., rapidity of progression).

Oncobiosis is also associated with disease recurrence. Kim et al. reported that when comparing patients with high and low risk of regional recurrence of breast cancer, bacterial pentose and glucuronate interconversions and *Enterococcus* were the main discriminating factors [95]. In support of these observations, antibiotic overdosing can increase the likelihood of disease recurrence [29].

Although little is known about the processes supporting disease recurrence, aldehyde dehydrogenase-1 (ALDH1) positive cancer stem cells are likely to have a supportive role. Bacterial metabolites (cadaverine and indolepropionic acid) produced by the healthy gut eubiome can reduce the proportions of ALDH1 + cancer stem cells in cultured cells [79, 81], leading to reduced recurrence and therapy resistance (Fig. 4). In breast cancer patients, the production of these metabolites is suppressed, supporting the expansion of ALDH1 + cancer stem cells [79, 81]. As noted earlier, widespread metabolic rearrangements were identified in the cancer tissue oncobiome [55–57, 59, 62, 67] and the gut oncobiome that encompass elements of Warburg metabolism [75, 78, 79, 81, 87, 89, 175]. Warburg-type metabolism may be a molecular mechanism through which oncobiosis supports ALDH1 + cancer stem cell formation.

## The role of the breast cancer oncobiome in chemotherapy

Neoadjuvation is a chemotherapeutic procedure aimed at shrinking tumor size to enable surgical excision. The microbiome metabolizes chemotherapeutic drugs, including those used in the (neoadjuvant) chemotherapy of breast cancer [270]. Hence, the microbiome can fundamentally change the pharmacokinetics and pharmacodynamics of these drugs (reviewed in [3, 4, 270, 271]). Not surprisingly, antibiotics modulate the pharmacokinetic and pharmacodynamic properties of chemotherapeutic drugs and the therapeutic outcome [272]. Pseudomonas *aeruginosa*-conditioned medium affected breast cancer cell proliferation and doxorubicin-induced cell death, highlighting the role of secreted bacterial metabolites and toxins [250], or possibly bacterial extracellular vesicles [90].

Most chemotherapy agents have antimicrobial effects and, thus, affect the microbiome [4]. In line with that concept, neoadjuvation changes the composition of the microbiome [86, 88, 250]. In terms of diversity, the reports show differences between compartments. Chiba et al. [250] reported decreased alpha diversity in breast microbiome in patients undergoing neoadjuvation compared to those undergoing surgery without neoadjuvation. With regard to the gut microbiome, Terrisse et al. [86] reported increases in alpha diversity between stool samples from the same patient undergoing neoadjuvant chemotherapy and after the completion of neoadjuvant chemotherapy. Beta diversity values were able to separate patients according to tumor size, grade, auxiliary node involvement, and TNM stage [86]. Wu and colleagues confirmed increases in alpha diversity of the gut microbiome upon neoadjuvation [88].

Chiba et al. [250] showed that *Pseudomonas* species increased, while *Prevotella* decreased in tumors undergoing neoadjuvation in the breast microbiome. Wu and colleagues [88] reported changes to *Bacteroidetes (g_Alistipes)*, *Firmicutes (g_Clostridium*, *g_Eubacterium*, *g_Bilophyla)*, and *Proteobacteria (g_Haemophylus)* in the gut microbiome. Terrisse et al. [86] reported a large set of biochemical pathways that were differentially regulated between the pre- and postneoadjuvant samples in the gut microbiome. Most of these pathways were involved in amino acid and nitrogen metabolism [86].

## Conclusions

The involvement of the microbiome in breast cancer is compelling. From a clinical perspective, it is important to understand that oncobiosis has to be preexisting [24]. In other words, dysbiosis is a factor that supports carcinogenesis but is unlikely to be the cause of the disease. The interactions between the different pools of the microbiome and breast cancer cells are multi-pronged. The microbiome plays a pivotal role in preventing the metastatic spread of breast cancer [24, 78, 79, 81, 175]. Furthermore, the oncobiome interacts with chemotherapy [86, 88, 250] and has an impact on disease recurrence [250]. Altogether, these data point out a possible role of managing the microbiome to provide novel leverage on breast cancer. These strategic considerations encompass the use of prebiotics [36], probiotics [37–45], diverse nutrition [46–49], and the careful use antibiotics to reduce the risk for breast cancer incidence and recurrence [22–33].

Microbiome changes with the stage, grade, or subtype of breast cancer. Hence, these stratifications should be assessed and patients in the study should be reported with all details. A good example of such a study design can be found in the paper of Plaza-Diaz and colleagues [273]. Along the same line, protocols for identifying external contaminations to the microbiome are very important [8, 69, 274], especially in samples with low biomass.

The microbiome possesses a large set of possible biomarkers; the different subtypes of breast cancer have different microbiomes that can be distinguished [70, 75]. Reports have identified possible markers both at the level of nucleic acids [69, 70, 72, 75] and at the protein level [81] in the breast and stool for the detection of the disease. Certain markers are quite promising (ROCs between 0.888 and 0.917). We would also like to point out a large number of review papers discussing diagnostic issues [275–279]. As mentioned earlier, microbiome biomarkers can potentially be used to assess the microbiome-chemotherapy interactions and be a useful source of data in personalized medicine. Taken together, the microbiome-breast cancer interactions have wide applicability from the clinical perspective that warrants future studies and applied discoveries.

## References

[1] Thomas RM, Jobin C (2015). The microbiome and cancer: Is the 'oncobiome' mirage real?. Trends in cancer.

[2] Kiss B, Mikó E, Sebő É, Toth J, Ujlaki G, Szabó J (2020). Oncobiosis and microbial metabolite signaling in pancreatic adenocarcinoma. Cancers (Basel).

[3] Sipos A, Ujlaki G, Mikó E, Maka E, Szabó J, Uray K (2021). The role of the microbiome in ovarian cancer: Mechanistic insights into oncobiosis and to bacterial metabolite signaling. Molecular Medicine.

[4] Miko E, Kovacs T, Sebo E, Toth J, Csonka T, Ujlaki G (2019). Microbiome-microbial metabolome-cancer cell interactions in breast cancer-familiar, but unexplored. Cells.

[5] Arthur JC, Jobin C (2011). The struggle within: Microbial influences on colorectal cancer. Inflammatory Bowel Diseases.

[6] Robinson KM, Crabtree J, Mattick JS, Anderson KE, Dunning Hotopp JC (2017). Distinguishing potential bacteria-tumor associations from contamination in a secondary data analysis of public cancer genome sequence data. Microbiome.

[7] Sepich-Poore GD, Zitvogel L, Straussman R, Hasty J, Wargo JA, Knight R (2021). The microbiome and human cancer. Science.

[8] Poore GD, Kopylova E, Zhu Q, Carpenter C, Fraraccio S, Wandro S (2020). Microbiome analyses of blood and tissues suggest cancer diagnostic approach. Nature.

[9] Dafni U, Tsourti Z, Alatsathianos I (2019). Breast Cancer Statistics in the European Union: Incidence and survival across European countries. Breast Care (Basel).

[10] Miller KD, Nogueira L, Mariotto AB, Rowland JH, Yabroff KR, Alfano CM (2019). Cancer treatment and survivorship statistics, 2019. CA: A Cancer Journal for Clinicians.

[11] Bleyer A, Welch HG (2012). Effect of three decades of screening mammography on breast-cancer incidence. New England Journal of Medicine.

[12] UK, C. R. (2019). UK breast cancer statistics. https://www.cancerresearchuk.org/health-professional/cancer-statistics/statistics-by-cancer-type/breast-cancer#heading-Zero. Accessed 2019. 03. 17. 2019.

[13] Armstrong N, Ryder S, Forbes C, Ross J, Quek RG (2019). A systematic review of the international prevalence of BRCA mutation in breast cancer. Clinical epidemiology.

[14] Senkus E, Kyriakides S, Ohno S, Penault-Llorca F, Poortmans P, Rutgers E (2015). Primary breast cancer: ESMO Clinical Practice Guidelines for diagnosis, treatment and follow-up. Annals of Oncology.

[15] Minicozzi P, Van Eycken L, Molinie F, Innos K, Guevara M, Marcos-Gragera R (2019). Comorbidities, age and period of diagnosis influence treatment and outcomes in early breast cancer. International Journal of Cancer.

[16] Breastcancer.org (2019). U.S. Breast Cancer Statistics https://www.breastcancer.org/symptoms/understand_bc/statistics. Accessed 2019. 03. 17. 2019.

[17] Cardoso F, Kyriakides S, Ohno S, Penault-Llorca F, Poortmans P, Rubio IT (2019). Early breast cancer: ESMO Clinical Practice Guidelines for diagnosis, treatment and follow-up. Annals of Oncology.

[18] Cardoso F, Paluch-Shimon S, Senkus E, Curigliano G, Aapro MS, André F (2020). 5th ESO-ESMO international consensus guidelines for advanced breast cancer (ABC 5). Annals of Oncology.

[19] de Azambuja E, Trapani D, Loibl S, Delaloge S, Senkus E, Criscitiello C (2020). ESMO management and treatment adapted recommendations in the COVID-19 era: Breast cancer. ESMO Open.

[20] Badve, S. S., Beitsch, P. D., Bose, S., Byrd, D. R., Chen, V. W., Connolly, J. L., et al. (2018). Breast.In *AJCC Cancer Staging Manual, Eighth Edition*. Chicago, IL

[21] Hill MJ, Goddard P, Williams RE (1971). Gut bacteria and aetiology of cancer of the breast. Lancet.

[22] Kirkup BM, McKee AM, Madgwick M, Price CA, Dreger SA, Makin KA (2020). Antibiotic-induced disturbances of the gut microbiota result in accelerated breast tumour growth via a mast cell-dependent pathway. bioRxiv.

[23] Kirkup B, McKee A, Makin K, Paveley J, Caim S, Alcon-Giner C (2019). Perturbation of the gut microbiota by antibiotics results in accelerated breast tumour growth and metabolic dysregulation. bioRxiv.

[24] Buchta Rosean C, Bostic RR, Ferey JCM, Feng TY, Azar FN, Tung KS (2019). Preexisting commensal dysbiosis is a host-intrinsic regulator of tissue inflammation and tumor cell dissemination in hormone receptor-positive breast cancer. Cancer Research.

[25] Velicer CM, Heckbert SR, Lampe JW, Potter JD, Robertson CA, Taplin SH (2004). Antibiotic use in relation to the risk of breast cancer. JAMA.

[26] Tamim HM, Hanley JA, Hajeer AH, Boivin JF, Collet JP (2008). Risk of breast cancer in relation to antibiotic use. Pharmacoepidemiology and Drug Safety.

[27] Sergentanis TN, Zagouri F, Zografos GC (2010). Is antibiotic use a risk factor for breast cancer? A meta-analysis. Pharmacoepidemiology and Drug Safety.

[28] Satram-Hoang S, Moran EM, Anton-Culver H, Burras RW, Heimann TM, Boggio I (2010). A pilot study of male breast cancer in the Veterans Affairs healthcare system. Journal of Environmental Pathology, Toxicology and Oncology.

[29] Wirtz HS, Buist DS, Gralow JR, Barlow WE, Gray S, Chubak J (2013). Frequent antibiotic use and second breast cancer events. Cancer Epidemiology, Biomarkers & Prevention.

[30] Elkrief A, Derosa L, Kroemer G, Zitvogel L, Routy B (2019). The negative impact of antibiotics on outcomes in cancer patients treated with immunotherapy: A new independent prognostic factor?. Annals of Oncology.

[31] Friedman GD, Oestreicher N, Chan J, Quesenberry CP, Udaltsova N, Habel LA (2006). Antibiotics and risk of breast cancer: Up to 9 years of follow-up of 2.1 million women. Cancer Epidemiology, Biomarkers & Prevention.

[32] Velicer CM, Heckbert SR, Rutter C, Lampe JW, Malone K (2006). Association between antibiotic use prior to breast cancer diagnosis and breast tumour characteristics (United States). Cancer Causes and Control.

[33] Simin J, Tamimi RM, Engstrand L, Callens S, Brusselaers N (2020). Antibiotic use and the risk of breast cancer: A systematic review and dose-response meta-analysis. Pharmacological Research.

[34] García Rodríguez LA, González-Pérez A (2005). Use of antibiotics and risk of breast cancer. American Journal of Epidemiology.

[35] Sørensen HT, Skriver MV, Friis S, McLaughlin JK, Blot WJ, Baron JA (2005). Use of antibiotics and risk of breast cancer: A population-based case–control study. British Journal of Cancer.

[36] Jiang Y, Fan L (2021). The effect of Poria cocos ethanol extract on the intestinal barrier function and intestinal microbiota in mice with breast cancer. Journal of Ethnopharmacology.

[37] Goubet A-G, Wheeler R, Fluckiger A, Qu B, Lemaître F, Iribarren K (2021). Multifaceted modes of action of the anticancer probiotic Enterococcus hirae. Cell Death & Differentiation.

[38] Aragon F, Carino S, Perdigon G, de Moreno de LeBlanc A (2014). The administration of milk fermented by the probiotic Lactobacillus casei CRL 431 exerts an immunomodulatory effect against a breast tumour in a mouse model. Immunobiology.

[39] Maroof H, Hassan ZM, Mobarez AM, Mohamadabadi MA (2012). Lactobacillus acidophilus could modulate the immune response against breast cancer in murine model. Journal of Clinical Immunology.

[40] Hassan Z, Mustafa S, Rahim RA, Isa NM (2016). Anti-breast cancer effects of live, heat-killed and cytoplasmic fractions of Enterococcus faecalis and Staphylococcus hominis isolated from human breast milk. In Vitro Cellular and Developmental Biology. Animal.

[41] Pourbaferani M, Modiri S, Norouzy A, Maleki H, Heidari M, Alidoust L (2021). A newly characterized potentially probiotic strain, Lactobacillus brevis MK05, and the Toxicity effects of its secretory proteins against MCF-7 breast cancer cells. Probiotics Antimicrob Proteins.

[42] Méndez Utz VE, Pérez Visñuk D, Perdigón G, de Moreno de LeBlanc A (2021). Milk fermented by Lactobacillus casei CRL431 administered as an immune adjuvant in models of breast cancer and metastasis under chemotherapy. Applied Microbiology and Biotechnology.

[43] Mendoza L (2019). Potential effect of probiotics in the treatment of breast cancer. Oncology Reviews.

[44] Ranjbar S, Seyednejad SA, Azimi H, Rezaeizadeh H, Rahimi R (2019). Emerging roles of probiotics in prevention and treatment of breast cancer: A comprehensive review of their therapeutic potential. Nutrition and Cancer.

[45] Ranjbar S, Seyednejad SA, Zakeri SE, Rezaeizadeh H, Rahimi R, Deol PK (2021). Probiotics for prophylaxis and management of breast cancer: Preclinical and clinical evidence. Probiotic Research in Therapeutics Applications in Cancers and Immunological Diseases.

[46] Lecuyer L, Dalle C, Lefevre-Arbogast S, Micheau P, Lyan B, Rossary A (2019). Diet-related metabolomic signature of long-term breast cancer risk using penalized regression: An exploratory study in the SU.VI.MAX cohort. Cancer Epidemiology, Biomarkers & Prevention.

[47] Newman, T. M., Vitolins, M. Z., & Cook, K. L. (2019). From the table to the tumor: The role of mediterranean and western dietary patterns in shifting microbial-mediated signaling to impact breast cancer risk. *Nutrients, 11*(11), 10.3390/nu11112565 10.3390/nu11112565 PMC6893457 31652909

[48] Guinter MA, McLain AC, Merchant AT, Sandler DP, Steck SE (2018). A dietary pattern based on estrogen metabolism is associated with breast cancer risk in a prospective cohort of postmenopausal women. International Journal of Cancer.

[49] Wu Y, Huang R, Wang M, Bernstein L, Bethea TN, Chen C (2021). Dairy foods, calcium, and risk of breast cancer overall and for subtypes defined by estrogen receptor status: A pooled analysis of 21 cohort studies. American Journal of Clinical Nutrition.

[50] Jones GS, Spencer Feigelson H, Falk RT, Hua X, Ravel J, Yu G (2019). Mammographic breast density and its association with urinary estrogens and the fecal microbiota in postmenopausal women. PLoS One..

[51] Wu AH, Tseng C, Vigen C, Yu Y, Cozen W, Garcia AA (2020). Gut microbiome associations with breast cancer risk factors and tumor characteristics: A pilot study. Breast Cancer Research and Treatment.

[52] Fruge AD, Van der Pol W, Rogers LQ, Morrow CD, Tsuruta Y, Demark-Wahnefried W (2018). Fecal Akkermansia muciniphila is associated with body composition and microbiota diversity in overweight and obese women with breast cancer participating in a presurgical weight loss trial. Journal of the Academy of Nutrition and Dietetics.

[53] Zhang X, Yang Y, Su J, Zheng X, Wang C, Chen S (2021). Age-related compositional changes and correlations of gut microbiome, serum metabolome, and immune factor in rats. Geroscience.

[54] Wang H, Altemus J, Niazi F, Green H, Calhoun BC, Sturgis C (2017). Breast tissue, oral and urinary microbiomes in breast cancer. Oncotarget.

[55] Urbaniak C, Gloor GB, Brackstone M, Scott L, Tangney M, Reid G (2016). The microbiota of breast tissue and its association with breast cancer. Applied and Environment Microbiology.

[56] Hieken TJ, Chen J, Hoskin TL, Walther-Antonio M, Johnson S, Ramaker S (2016). The microbiome of aseptically collected human breast tissue in benign and malignant disease. Science and Reports.

[57] Chan AA, Bashir M, Rivas MN, Duvall K, Sieling PA, Pieber TR (2016). Characterization of the microbiome of nipple aspirate fluid of breast cancer survivors. Science and Reports.

[58] Meng S, Chen B, Yang J, Wang J, Zhu D, Meng Q (2018). Study of microbiomes in aseptically collected samples of human breast tissue using needle biopsy and the potential role of in situ tissue microbiomes for promoting malignancy. Frontiers in Oncology.

[59] Smith A, Pierre JF, Makowski L, Tolley E, Lyn-Cook B, Lu L (2019). Distinct microbial communities that differ by race, stage, or breast-tumor subtype in breast tissues of non-Hispanic Black and non-Hispanic White women. Science and Reports.

[60] Tzeng A, Sangwan N, Jia M, Liu C-C, Keslar KS, Downs-Kelly E (2021). Human breast microbiome correlates with prognostic features and immunological signatures in breast cancer. Genome Medicine.

[61] Costantini L, Magno S, Albanese D, Donati C, Molinari R, Filippone A (2018). Characterization of human breast tissue microbiota from core needle biopsies through the analysis of multi hypervariable 16S-rRNA gene regions. Science and Reports.

[62] Klann E, Williamson JM, Tagliamonte MS, Ukhanova M, Asirvatham JR, Chim H (2020). Microbiota composition in bilateral healthy breast tissue and breast tumors. Cancer Causes and Control.

[63] Thyagarajan S, Zhang Y, Thapa S, Allen MS, Phillips N, Chaudhary P (2020). Comparative analysis of racial differences in breast tumor microbiome. Science and Reports.

[64] Banerjee S, Wei Z, Tan F, Peck KN, Shih N, Feldman M (2015). Distinct microbiological signatures associated with triple negative breast cancer. Science and Reports.

[65] Banerjee S, Tian T, Wei Z, Shih N, Feldman MD, Peck KN (2018). Distinct microbial signatures associated with different breast cancer types. Frontiers in Microbiology.

[66] Xuan C, Shamonki JM, Chung A, Dinome ML, Chung M, Sieling PA (2014). Microbial dysbiosis is associated with human breast cancer. PLoS One.

[67] Nejman D, Livyatan I, Fuks G, Gavert N, Zwang Y, Geller LT (2020). The human tumor microbiome is composed of tumor type-specific intracellular bacteria. Science.

[68] Thompson KJ, Ingle JN, Tang X, Chia N, Jeraldo PR, Walther-Antonio MR (2017). A comprehensive analysis of breast cancer microbiota and host gene expression. PLoS One.

[69] Hogan G, Eckenberger J, Narayanen N, Walker SP, Claesson MJ, Corrigan M (2021). Biopsy bacterial signature can predict patient tissue malignancy. Scientific Reports.

[70] Banerjee S, Wei Z, Tian T, Bose D, Shih NNC, Feldman MD (2021). Prognostic correlations with the microbiome of breast cancer subtypes. Cell Death & Disease.

[71] Giallourou N, Urbaniak C, Puebla-Barragan S, Vorkas PA, Swann JR, Reid G (2021). Characterizing the breast cancer lipidome and its interaction with the tissue microbiota. Communications Biology.

[72] Mao AW, Barck H, Young J, Paley A, Mao J, Chang H (2021). Identification of a novel cancer microbiome signature for predicting prognosis of human breast cancer patients. Clinical and Translational Oncology.

[73] Goedert JJ, Jones G, Hua X, Xu X, Yu G, Flores R (2015). Investigation of the association between the fecal microbiota and breast cancer in postmenopausal women: A population-based case-control pilot study. J Natl Cancer Inst..

[74] Byrd, D. A., Vogtmann, E., Wu, Z., Han, Y., Wan, Y., Clegg-Lamptey, J.-N., et al. (2021). Associations of fecal microbial profiles with breast cancer and non-malignant breast disease in the Ghana Breast Health Study. *International Journal of Cancer, n/a*(n/a), 10.1002/ijc.33473 10.1002/ijc.33473 PMC8386185 33460452

[75] Zhu J, Liao M, Yao Z, Liang W, Li Q, Liu J (2018). Breast cancer in postmenopausal women is associated with an altered gut metagenome. Microbiome..

[76] Howe C, Kim SJ, Mitchell J, Im E, Kim YS, Kim YS (2018). Differential expression of tumor-associated genes and altered gut microbiome with decreased Akkermansia muciniphila confer a tumor-preventive microenvironment in intestinal epithelial Pten-deficient mice. Biochimica et Biophysica Acta, Molecular Basis of Disease.

[77] Luu TH, Michel C, Bard JM, Dravet F, Nazih H, Bobin-Dubigeon C (2017). Intestinal proportion of Blautia sp. is associated with clinical stage and histoprognostic grade in patients with early-stage breast cancer. Nutrition and Cancer.

[78] Miko E, Vida A, Kovacs T, Ujlaki G, Trencsenyi G, Marton J (2018). Lithocholic acid, a bacterial metabolite reduces breast cancer cell proliferation and aggressiveness. Biochimica et Biophysica Acta.

[79] Kovács T, Mikó E, Vida A, Sebő É, Toth J, Csonka T (2019). Cadaverine, a metabolite of the microbiome, reduces breast cancer aggressiveness through trace amino acid receptors. Science and Reports.

[80] Sári Z, Kovács T, Csonka T, Török M, Sebő É, Toth J (2020). Fecal expression of E. coli lysine decarboxylase (LdcC) is downregulated in E-cadherin negative lobular breast carcinoma. Physiology International.

[81] Sári Z, Mikó E, Kovács T, Jankó L, Csonka T, Sebő E (2020). Indolepropionic acid, a metabolite of the microbiome, has cytostatic properties in breast cancer by activating AHR and PXR receptors and inducing oxidative stress. Cancers (Basel).

[82] Murray WR, Blackwood A, Calman KC, MacKay C (1980). Faecal bile acids and clostridia in patients with breast cancer. British Journal of Cancer.

[83] Goedert JJ, Hua X, Bielecka A, Okayasu I, Milne GL, Jones GS (2018). Postmenopausal breast cancer and oestrogen associations with the IgA-coated and IgA-noncoated faecal microbiota. British Journal of Cancer.

[84] Bobin-Dubigeon, C., Luu, H. T., Leuillet, S., Lavergne, S. N., Carton, T., Le Vacon, F., et al. (2021). Faecal microbiota composition varies between patients with breast cancer and healthy women: A comparative case-control study. *Nutrients, 13*(8), 10.3390/nu13082705. 10.3390/nu13082705 PMC8399700 34444865

[85] Horigome A, Okubo R, Hamazaki K, Kinoshita T, Katsumata N, Uezono Y (2019). Association between blood omega-3 polyunsaturated fatty acids and the gut microbiota among breast cancer survivors. Benef Microbes.

[86] Terrisse S, Derosa L, Iebba V, Ghiringhelli F, Vaz-Luis I, Kroemer G (2021). Intestinal microbiota influences clinical outcome and side effects of early breast cancer treatment. Cell Death & Differentiation.

[87] Hou MF, Ou-Yang F, Li CL, Chen FM, Chuang CH, Kan JY (2021). Comprehensive profiles and diagnostic value of menopausal-specific gut microbiota in premenopausal breast cancer. Experimental & Molecular Medicine.

[88] Wu, A. H., Vigen, C., Tseng, C., Garcia, A. A., & Spicer, D. (2021). Effect of chemotherapy and weight change on the gut microbiome of breast cancer patients during the first year of treatment. *Research Square*, https://www.researchsquare.com/article/rs-970564/v970561. 10.2147/BCTT.S305486 PMC9747861 36532254

[89] Yang P, Wang Z, Peng Q, Lian W, Chen D (2021). Comparison of the gut microbiota in patients with benign and malignant breast tumors: A pilot study. Evol Bioinform Online.

[90] An, J., Kim, J. B., Yang, E. Y., Kim, H. O., Lee, W.-H., Yang, J., et al. (2021). Bacterial extracellular vesicles affect endocrine therapy in MCF7 cells. *Medicine, 100*(18). 10.1097/MD.0000000000025835 PMC8104188 33950995

[91] Bose D, Banerjee S, Singh RK, Wise LM, Robertson ES (2020). Vascular endothelial growth factor encoded by Parapoxviruses can regulate metabolism and survival of triple negative breast cancer cells. Cell Death & Disease.

[92] Zhang N, Ma ZP, Wang J, Bai HL, Li YX, Sun Q (2016). Human papillomavirus infection correlates with inflammatory Stat3 signaling activity and IL-17 expression in patients with breast cancer. American Journal of Translational Research.

[93] Rao Malla R, Marni R, Kumari S, Chakraborty A, Lalitha P (2021). Microbiome assisted tumor microenvironment: Emerging target of breast cancer. Clinical Breast Cancer.

[94] Miko E, Vida A, Bai P (2016). Translational aspects of the microbiome-to be exploited. Cell Biology and Toxicology.

[95] Kim, H. E., Kim, J., Maeng, S., Oh, B., Hwang, K. T., & Kim, B. S. (2021). Microbiota of breast tissue and its potential association with regional recurrence of breast cancer in Korean women. *Journal of Microbiology and Biotechnology, 31*(11), 10.4014/jmb.2106.06039. 10.4014/jmb.2106.06039 PMC9705848 34584037

[96] Urbaniak C, Cummins J, Brackstone M, Macklaim JM, Gloor GB, Baban CK (2014). Microbiota of human breast tissue. Applied and Environment Microbiology.

[97] Kovács P, Csonka T, Kovács T, Sári Z, Ujlaki G, Sipos A (2019). Lithocholic acid, a metabolite of the microbiome, increases oxidative stress in breast cancer. Cancers (Basel).

[98] Karihtala P, Kauppila S, Soini Y, Arja Jukkola V (2011). Oxidative stress and counteracting mechanisms in hormone receptor positive, triple-negative and basal-like breast carcinomas. BMC Cancer.

[99] Smolková K, Mikó E, Kovács T, Leguina-Ruzzi A, Sipos A, Bai P (2020). NRF2 in regulating cancer metabolism. Antioxidants & Redox Signaling.

[100] Reyes AM, Pedre B, De Armas MI, Tossounian MA, Radi R, Messens J (2018). Chemistry and redox biology of mycothiol. Antioxidants & Redox Signaling.

[101] Nougayrède JP, Homburg S, Taieb F, Boury M, Brzuszkiewicz E, Gottschalk G (2006). Escherichia coli induces DNA double-strand breaks in eukaryotic cells. Science.

[102] Cuevas-Ramos G, Petit CR, Marcq I, Boury M, Oswald E, Nougayrède JP (2010). Escherichia coli induces DNA damage in vivo and triggers genomic instability in mammalian cells. Proceedings of the National Academy of Sciences of the United States of America.

[103] Pavlides S, Whitaker-Menezes D, Castello-Cros R, Flomenberg N, Witkiewicz AK, Frank PG (2009). The reverse Warburg effect: Aerobic glycolysis in cancer associated fibroblasts and the tumor stroma. Cell Cycle.

[104] Migneco G, Whitaker-Menezes D, Chiavarina B, Castello-Cros R, Pavlides S, Pestell RG (2010). Glycolytic cancer associated fibroblasts promote breast cancer tumor growth, without a measurable increase in angiogenesis: Evidence for stromal-epithelial metabolic coupling. Cell Cycle.

[105] Bonuccelli G, Whitaker-Menezes D, Castello-Cros R, Pavlides S, Pestell RG, Fatatis A (2010). The reverse Warburg effect: Glycolysis inhibitors prevent the tumor promoting effects of caveolin-1 deficient cancer associated fibroblasts. Cell Cycle.

[106] Bonuccelli G, Tsirigos A, Whitaker-Menezes D, Pavlides S, Pestell RG, Chiavarina B (2010). Ketones and lactate "fuel" tumor growth and metastasis: Evidence that epithelial cancer cells use oxidative mitochondrial metabolism. Cell Cycle.

[107] Kim S, Kim DH, Jung WH, Koo JS (2013). Metabolic phenotypes in triple-negative breast cancer. Tumour Biology.

[108] Choi J, Kim DH, Jung WH, Koo JS (2013). Metabolic interaction between cancer cells and stromal cells according to breast cancer molecular subtype. Breast Cancer Research.

[109] Martinez-Outschoorn U, Sotgia F, Lisanti MP (2014). Tumor microenvironment and metabolic synergy in breast cancers: Critical importance of mitochondrial fuels and function. Seminars in Oncology.

[110] Gang BP, Dilda PJ, Hogg PJ, Blackburn AC (2014). Targeting of two aspects of metabolism in breast cancer treatment. Cancer Biology and Therapy.

[111] Fodor T, Szanto M, Abdul-Rahman O, Nagy L, Der A, Kiss B (2016). Combined treatment of MCF-7 Cells with AICAR and methotrexate, arrests cell cycle and reverses Warburg metabolism through AMP-activated protein kinase (AMPK) and FOXO1. PLoS One.

[112] Elia I, Schmieder R, Christen S, Fendt SM (2016). Organ-specific cancer metabolism and its potential for therapy. Handbook of Experimental Pharmacology.

[113] Martinez-Outschoorn UE, Lisanti MP, Sotgia F (2014). Catabolic cancer-associated fibroblasts transfer energy and biomass to anabolic cancer cells, fueling tumor growth. Seminars in Cancer Biology.

[114] Ligorio F, Pellegrini I, Castagnoli L, Vingiani A, Lobefaro R, Zattarin E (2021). Targeting lipid metabolism is an emerging strategy to enhance the efficacy of anti-HER2 therapies in HER2-positive breast cancer. Cancer Letters.

[115] Guo, R., Chen, Y., Borgard, H., Jijiwa, M., Nasu, M., He, M., et al. (2020). The function and mechanism of lipid molecules and their roles in the diagnosis and prognosis of breast cancer. *Molecules, 25*(20), 10.3390/molecules25204864. 10.3390/molecules25204864 PMC7588012 33096860

[116] Nazih, H., & Bard, J. M. (2020). Cholesterol, oxysterols and LXRs in breast cancer pathophysiology. *International Journal of Molecular Sciences, 21*(4), 10.3390/ijms21041356. 10.3390/ijms21041356 PMC7072989 32079340

[117] Dabek M, McCrae SI, Stevens VJ, Duncan SH, Louis P (2008). Distribution of beta-glucosidase and beta-glucuronidase activity and of beta-glucuronidase gene gus in human colonic bacteria. FEMS Microbiology Ecology.

[118] McIntosh FM, Maison N, Holtrop G, Young P, Stevens VJ, Ince J (2012). Phylogenetic distribution of genes encoding beta-glucuronidase activity in human colonic bacteria and the impact of diet on faecal glycosidase activities. Environmental Microbiology.

[119] Gloux K, Berteau O, El Oumami H, Beguet F, Leclerc M, Dore J (2011). A metagenomic beta-glucuronidase uncovers a core adaptive function of the human intestinal microbiome. Proceedings of the National Academy of Sciences of the United States of America.

[120] Kwa M, Plottel CS, Blaser MJ, Adams S (2016). The Intestinal microbiome and estrogen receptor-positive female breast cancer. Journal of the National Cancer Institute.

[121] Flores R, Shi J, Fuhrman B, Xu X, Veenstra TD, Gail MH (2012). Fecal microbial determinants of fecal and systemic estrogens and estrogen metabolites: A cross-sectional study. Journal of Translational Medicine.

[122] Fuhrman BJ, Feigelson HS, Flores R, Gail MH, Xu X, Ravel J (2014). Associations of the fecal microbiome with urinary estrogens and estrogen metabolites in postmenopausal women. Journal of Clinical Endocrinology and Metabolism.

[123] Ervin SM, Li H, Lim L, Roberts LR, Liang X, Mani S (2019). Gut microbial beta-glucuronidases reactivate estrogens as components of the estrobolome that reactivate estrogens. Journal of Biological Chemistry.

[124] Komorowski AS, Pezo RC (2020). Untapped "-omics": The microbial metagenome, estrobolome, and their influence on the development of breast cancer and response to treatment. Breast Cancer Research and Treatment.

[125] Sharon G, Garg N, Debelius J, Knight R, Dorrestein PC, Mazmanian SK (2014). Specialized metabolites from the microbiome in health and disease. Cell Metabolism.

[126] Wilmanski T, Rappaport N, Earls JC, Magis AT, Manor O, Lovejoy J (2019). Blood metabolome predicts gut microbiome alpha-diversity in humans. Nature Biotechnology.

[127] Kumari S, Malla RR (2020). Recent advances in metabolomics of triple negative breast cancer. The Breast Journal.

[128] Tenori L, Oakman C, Morris PG, Gralka E, Turner N, Cappadona S (2015). Serum metabolomic profiles evaluated after surgery may identify patients with oestrogen receptor negative early breast cancer at increased risk of disease recurrence. Results from a retrospective study. Molecular Oncology.

[129] Jobard E, Pontoizeau C, Blaise BJ, Bachelot T, Elena-Herrmann B, Trédan O (2014). A serum nuclear magnetic resonance-based metabolomic signature of advanced metastatic human breast cancer. Cancer Letters.

[130] Asiago VM, Alvarado LZ, Shanaiah N, Gowda GA, Owusu-Sarfo K, Ballas RA (2010). Early detection of recurrent breast cancer using metabolite profiling. Cancer Research.

[131] Slupsky CM, Steed H, Wells TH, Dabbs K, Schepansky A, Capstick V (2010). Urine metabolite analysis offers potential early diagnosis of ovarian and breast cancers. Clinical Cancer Research.

[132] Harada-Shoji N, Soga T, Tada H, Miyashita M, Harada M, Watanabe G (2019). A metabolic profile of routine needle biopsies identified tumor type specific metabolic signatures for breast cancer stratification: A pilot study. Metabolomics.

[133] Yu LC, Wei SC, Li YH, Lin PY, Chang XY, Weng JP (2021). Invasive pathobionts contribute to colon cancer initiation by counterbalancing epithelial antimicrobial responses. Cellular and Molecular Gastroenterology and Hepatology.

[134] Kuo WT, Lee TC, Yu LC (2016). Eritoran suppresses colon cancer by altering a functional balance in toll-like receptors that bind lipopolysaccharide. Cancer Research.

[135] Arthur JC, Perez-Chanona E, Mühlbauer M, Tomkovich S, Uronis JM, Fan TJ (2012). Intestinal inflammation targets cancer-inducing activity of the microbiota. Science.

[136] Micevych PE, Kelly MJ (2012). Membrane estrogen receptor regulation of hypothalamic function. Neuroendocrinology.

[137] Soltysik K, Czekaj P (2013). Membrane estrogen receptors - is it an alternative way of estrogen action?. Journal of Physiology and Pharmacology.

[138] Radde BN, Ivanova MM, Mai HX, Salabei JK, Hill BG, Klinge CM (2015). Bioenergetic differences between MCF-7 and T47D breast cancer cells and their regulation by oestradiol and tamoxifen. The Biochemical Journal.

[139] Radde BN, Ivanova MM, Mai HX, Alizadeh-Rad N, Piell K, Van Hoose P (2016). Nuclear respiratory factor-1 and bioenergetics in tamoxifen-resistant breast cancer cells. Experimental Cell Research.

[140] Sotgia F, Lisanti MP (2017). Mitochondrial mRNA transcripts predict overall survival, tumor recurrence and progression in serous ovarian cancer: Companion diagnostics for cancer therapy. Oncotarget.

[141] Gandhi N, Das GM (2019). Metabolic reprogramming in breast cancer and its therapeutic implications. Cells.

[142] Zacksenhaus E, Shrestha M, Liu JC, Vorobieva I, Chung PED, Ju Y (2017). Mitochondrial OXPHOS induced by RB1 deficiency in breast cancer: Implications for anabolic metabolism, stemness, and metastasis. Trends in Cancer.

[143] Maximov PY, Abderrahman B, Curpan RF, Hawsawi YM, Fan P, Jordan VC (2018). A unifying biology of sex steroid-induced apoptosis in prostate and breast cancers. Endocrine-Related Cancer.

[144] Al-Howail HA, Hakami HA, Al-Otaibi B, Al-Mazrou A, Daghestani MH, Al-Jammaz I (2016). PAC down-regulates estrogen receptor alpha and suppresses epithelial-to-mesenchymal transition in breast cancer cells. BMC Cancer.

[145] Bouris P, Skandalis SS, Piperigkou Z, Afratis N, Karamanou K, Aletras AJ (2015). Estrogen receptor alpha mediates epithelial to mesenchymal transition, expression of specific matrix effectors and functional properties of breast cancer cells. Matrix Biology.

[146] Kulkoyluoglu-Cotul E, Arca A, Madak-Erdogan Z (2019). Crosstalk between estrogen signaling and breast cancer metabolism. Trends in Endocrinology and Metabolism.

[147] Derrien M, Vaughan EE, Plugge CM, de Vos WM (2004). Akkermansia muciniphila gen. nov., sp. nov., a human intestinal mucin-degrading bacterium. International Journal of Systematic and Evolutionary Microbiology.

[148] Louis P, Young P, Holtrop G, Flint HJ (2010). Diversity of human colonic butyrate-producing bacteria revealed by analysis of the butyryl-CoA:Acetate CoA-transferase gene. Environmental Microbiology.

[149] Reichardt N, Duncan SH, Young P, Belenguer A, McWilliam Leitch C, Scott KP (2014). Phylogenetic distribution of three pathways for propionate production within the human gut microbiota. ISME Journal.

[150] Zhao C, Dong H, Zhang Y, Li Y (2019). Discovery of potential genes contributing to the biosynthesis of short-chain fatty acids and lactate in gut microbiota from systematic investigation in E. coli. NPJ Biofilms and Microbiomes.

[151] Jin UH, Cheng Y, Park H, Davidson LA, Callaway ES, Chapkin RS (2017). Short chain fatty acids enhance aryl hydrocarbon (Ah) responsiveness in mouse colonocytes and Caco-2 human colon cancer cells. Science and Reports.

[152] Shimazu T, Hirschey MD, Newman J, He W, Shirakawa K, Le Moan N (2013). Suppression of oxidative stress by beta-hydroxybutyrate, an endogenous histone deacetylase inhibitor. Science.

[153] Priyadarshini M, Kotlo KU, Dudeja PK, Layden BT (2018). Role of short chain fatty acid receptors in intestinal physiology and pathophysiology. Comprehensive Physiology.

[154] Arpaia N, Campbell C, Fan X, Dikiy S, van der Veeken J, deRoos P (2013). Metabolites produced by commensal bacteria promote peripheral regulatory T-cell generation. Nature.

[155] Tan J, McKenzie C, Potamitis M, Thorburn AN, Mackay CR, Macia L (2014). The role of short-chain fatty acids in health and disease. Advances in Immunology.

[156] Schulthess J, Pandey S, Capitani M, Rue-Albrecht KC, Arnold I, Franchini F (2019). The short chain fatty acid butyrate imprints an antimicrobial program in macrophages. Immunity.

[157] Salimi V, Shahsavari Z, Safizadeh B, Hosseini A, Khademian N, Tavakoli-Yaraki M (2017). Sodium butyrate promotes apoptosis in breast cancer cells through reactive oxygen species (ROS) formation and mitochondrial impairment. Lipids in Health and Disease.

[158] Rodrigues MF, Carvalho E, Pezzuto P, Rumjanek FD, Amoedo ND (2015). Reciprocal modulation of histone deacetylase inhibitors sodium butyrate and trichostatin A on the energy metabolism of breast cancer cells. Journal of Cellular Biochemistry.

[159] Aries V, Hill MJ (1970). Degradation of steroids by intestinal bacteria. I. Deconjugation of bile salts. Biochimica et Biophysica Acta - Bioenergetics.

[160] Jarocki P, Targoński Z (2013). Genetic diversity of bile salt hydrolases among human intestinal bifidobacteria. Current Microbiology.

[161] Oh HK, Lee JY, Lim SJ, Kim MJ, Kim GB, Kim JH (2008). Molecular cloning and characterization of a bile salt hydrolase from Lactobacillus acidophilus PF01. Journal of Microbiology and Biotechnology.

[162] Marion S, Desharnais L, Studer N, Dong Y, Notter MD, Poudel S (2020). Biogeography of microbial bile acid transformations along the murine gut. Journal of Lipid Research.

[163] Jones BV, Begley M, Hill C, Gahan CG, Marchesi JR (2008). Functional and comparative metagenomic analysis of bile salt hydrolase activity in the human gut microbiome. Proceedings of the National Academy of Sciences of the United States of America.

[164] Gerard P (2013). Metabolism of cholesterol and bile acids by the gut microbiota. Pathogens.

[165] Ridlon JM, Devendran S, Alves JM, Doden H, Wolf PG, Pereira GV (2020). The 'in vivo lifestyle' of bile acid 7α-dehydroxylating bacteria: Comparative genomics, metatranscriptomic, and bile acid metabolomics analysis of a defined microbial community in gnotobiotic mice x. Gut Microbes.

[166] Vital M, Rud T, Rath S, Pieper DH, Schlüter D (2019). Diversity of bacteria exhibiting bile acid-inducible 7α-dehydroxylation genes in the human gut. Computational and Structural Biotechnology Journal.

[167] Ridlon JM, Harris SC, Bhowmik S, Kang DJ, Hylemon PB (2016). Consequences of bile salt biotransformations by intestinal bacteria. Gut Microbes.

[168] Kawamata Y, Fujii R, Hosoya M, Harada M, Yoshida H, Miwa M (2003). A G protein-coupled receptor responsive to bile acids. Journal of Biological Chemistry.

[169] Makishima M, Okamoto AY, Repa JJ, Tu H, Learned RM, Luk A (1999). Identification of a nuclear receptor for bile acids. Science.

[170] Zhang Y, Hagedorn CH, Wang L (2011). Role of nuclear receptor SHP in metabolism and cancer. Biochimica et Biophysica Acta - Molecular Basis of Disease.

[171] Luu TH, Bard JM, Carbonnelle D, Chaillou C, Huvelin JM, Bobin-Dubigeon C (2018). Lithocholic bile acid inhibits lipogenesis and induces apoptosis in breast cancer cells. Cellular Oncology (Dordrecht).

[172] Seiler N (2004). Catabolism of polyamines. Amino Acids.

[173] de las Rivas B, Marcobal A, Carrascosa AV, Munoz R (2006). PCR detection of foodborne bacteria producing the biogenic amines histamine, tyramine, putrescine, and cadaverine. Journal of Food Protection.

[174] Vattai A, Akyol E, Kuhn C, Hofmann S, Heidegger H, von Koch F (2017). Increased trace amine-associated receptor 1 (TAAR1) expression is associated with a positive survival rate in patients with breast cancer. Journal of cancer research and clinical oncology.

[175] Sári Z, Mikó E, Kovács T, Boratkó A, Ujlaki G, Jankó L (2020). Indoxylsulfate, a metabolite of the microbiome, has cytostatic effects in breast cancer via activation of AHR and PXR receptors and induction of oxidative stress. Cancers (Basel).

[176] Roager HM, Licht TR (2018). Microbial tryptophan catabolites in health and disease. Nature Communications.

[177] Bertani B, Ruiz N (2018). Function and biogenesis of lipopolysaccharides. Ecosal Plus.

[178] Erwin, A. L. (2016). Antibacterial drug discovery targeting the lipopolysaccharide biosynthetic enzyme LpxC. *Cold Spring Harbor Perspectives in Medicine, 6*(7), 10.1101/cshperspect.a025304. 10.1101/cshperspect.a025304 PMC4930914 27235477

[179] Garrett TA, Que NL, Raetz CR (1998). Accumulation of a lipid A precursor lacking the 4'-phosphate following inactivation of the Escherichia coli lpxK gene. Journal of Biological Chemistry.

[180] Lu Y-C, Yeh W-C, Ohashi PS (2008). LPS/TLR4 signal transduction pathway. Cytokine.

[181] Fried S, Tosun S, Troost G, Keil S, Zaenker KS, Dittmar T (2016). Lipopolysaccharide (LPS) promotes apoptosis in human breast epithelial × breast cancer hybrids, but not in parental cells. PLoS One.

[182] Li J, Yin J, Shen W, Gao R, Liu Y, Chen Y (2017). TLR4 promotes breast cancer metastasis via Akt/GSK3β/β-catenin pathway upon LPS stimulation. Anatomical Record (Hoboken).

[183] Yang H, Wang B, Wang T, Xu L, He C, Wen H (2014). Toll-like receptor 4 prompts human breast cancer cells invasiveness via lipopolysaccharide stimulation and is overexpressed in patients with lymph node metastasis x. PLoS One.

[184] Zheng L, Lin Y, Lu S, Zhang J, Bogdanov M (2017). Biogenesis, transport and remodeling of lysophospholipids in Gram-negative bacteria. Biochimica et biophysica acta Molecular and cell biology of lipids.

[185] Lin M-E, Herr DR, Chun J (2010). Lysophosphatidic acid (LPA) receptors: Signaling properties and disease relevance. Prostaglandins & other lipid mediators.

[186] Ye X (2008). Lysophospholipid signaling in the function and pathology of the reproductive system. Human Reproduction Update.

[187] Imagawa W, Bandyopadhyay GK, Nandi S (1995). Analysis of the proliferative response to lysophosphatidic acid in primary cultures of mammary epithelium: Differences between normal and tumor cells. Experimental Cell Research.

[188] Stadler CR, Knyazev P, Bange J, Ullrich A (2006). FGFR4 GLY388 isotype suppresses motility of MDA-MB-231 breast cancer cells by EDG-2 gene repression. Cellular Signalling.

[189] Dorfleutner A, Stehlik C, Zhang J, Gallick GE, Flynn DC (2007). AFAP-110 is required for actin stress fiber formation and cell adhesion in MDA-MB-231 breast cancer cells. Journal of Cellular Physiology.

[190] Faïs, T., Delmas, J., Barnich, N., Bonnet, R., & Dalmasso, G. (2018). Colibactin: More than a new bacterial toxin. *Toxins, 10*(4), 10.3390/toxins10040151. 10.3390/toxins10040151 PMC5923317 29642622

[191] Ridlon JM, Kang DJ, Hylemon PB (2006). Bile salt biotransformations by human intestinal bacteria. Journal of Lipid Research.

[192] Ridlon JM, Wolf PG, Gaskins HR (2016). Taurocholic acid metabolism by gut microbes and colon cancer. Gut Microbes.

[193] Tsuei J, Chau T, Mills D, Wan YJ (2014). Bile acid dysregulation, gut dysbiosis, and gastrointestinal cancer. Experimental Biology and Medicine (Maywood, N.J.).

[194] Merritt ME, Donaldson JR (2009). Effect of bile salts on the DNA and membrane integrity of enteric bacteria. Journal of Medical Microbiology.

[195] Garcia-Quintanilla M, Prieto AI, Barnes L, Ramos-Morales F, Casadesus J (2006). Bile-induced curing of the virulence plasmid in Salmonella enterica serovar Typhimurium. Journal of Bacteriology.

[196] Prieto AI, Ramos-Morales F, Casadesus J (2006). Repair of DNA damage induced by bile salts in Salmonella enterica. Genetics.

[197] Prieto AI, Ramos-Morales F, Casadesus J (2004). Bile-induced DNA damage in Salmonella enterica. Genetics.

[198] Kandell RL, Bernstein C (1991). Bile salt/acid induction of DNA damage in bacterial and mammalian cells: Implications for colon cancer. Nutrition and Cancer.

[199] Schaffler H, Breitruck A (2018). Clostridium difficile - from colonization to infection. Frontiers in Microbiology.

[200] Sorg JA, Sonenshein AL (2010). Inhibiting the initiation of Clostridium difficile spore germination using analogs of chenodeoxycholic acid, a bile acid. Journal of Bacteriology.

[201] Slocum MM, Sittig KM, Specian RD, Deitch EA (1992). Absence of intestinal bile promotes bacterial translocation. American Surgeon.

[202] Mukherji R, Prabhune A (2015). Possible correlation between bile salt hydrolysis and AHL deamidation: Staphylococcus epidermidis RM1, a potent quorum quencher and bile salt Hydrolase Producer. Applied Biochemistry and Biotechnology.

[203] Walawalkar, Y. D., Vaidya, Y., & Nayak, V. (2016). Response of Salmonella typhi to bile-generated oxidative stress: Implication of quorum sensing and persister cell populations. *Pathogens and Disease, 74*(8), 10.1093/femspd/ftw090. 10.1093/femspd/ftw090 27609462

[204] Javitt NB, Budai K, Miller DG, Cahan AC, Raju U, Levitz M (1994). Breast-gut connection: Origin of chenodeoxycholic acid in breast cyst fluid. Lancet.

[205] Tang W, Putluri V, Ambati CR, Dorsey TH, Putluri N, Ambs S (2019). Liver- and microbiome-derived bile acids accumulate in human breast tumors and inhibit growth and improve patient survival. Clinical Cancer Research.

[206] Raju U, Levitz M, Javitt NB (1990). Bile acids in human breast cyst fluid: The identification of lithocholic acid. Journal of Clinical Endocrinology and Metabolism.

[207] Tang X, Lin CC, Spasojevic I, Iversen ES, Chi JT, Marks JR (2014). A joint analysis of metabolomics and genetics of breast cancer. Breast Cancer Research.

[208] Luo C, Zhang X, He Y, Chen H, Liu M, Wang H (2021). A pseudo-targeted metabolomics study based on serum bile acids profiling for the differential diagnosis of benign and malignant breast lesions. Steroids.

[209] Costarelli V, Sanders TA (2002). Plasma deoxycholic acid concentration is elevated in postmenopausal women with newly diagnosed breast cancer. European Journal of Clinical Nutrition.

[210] Costarelli V, Sanders TA (2002). Plasma bile acids and risk of breast cancer. IARC Scientific Publications.

[211] Macdonald IA, Hill MJ (1979). The inability of nuclear dehydrogenating clostridia to oxidize bile salt hydroxyl groups. Experientia.

[212] Sreekanth V, Bansal S, Motiani RK, Kundu S, Muppu SK, Majumdar TD (2013). Design, synthesis, and mechanistic investigations of bile acid-tamoxifen conjugates for breast cancer therapy. Bioconjugate Chemistry.

[213] Yokoyama MT, Carlson JR (1979). Microbial metabolites of tryptophan in the intestinal tract with special reference to skatole. American Journal of Clinical Nutrition.

[214] Danaceau JP, Anderson GM, McMahon WM, Crouch DJ (2003). A liquid chromatographic-tandem mass spectrometric method for the analysis of serotonin and related indoles in human whole blood. Journal of Analytical Toxicology.

[215] Rosas HD, Doros G, Bhasin S, Thomas B, Gevorkian S, Malarick K (2015). A systems-level "misunderstanding": The plasma metabolome in Huntington's disease. Annals of Clinical Translational Neurology.

[216] Lin C-N, Wu IW, Huang Y-F, Peng S-Y, Huang Y-C, Ning H-C (2019). Measuring serum total and free indoxyl sulfate and p-cresyl sulfate in chronic kidney disease using UPLC-MS/MS. Journal of Food and Drug Analysis.

[217] DeMoss RD, Moser K (1969). Tryptophanase in diverse bacterial species. Journal of Bacteriology.

[218] Ma Q, Zhang X, Qu Y (2018). Biodegradation and biotransformation of indole: Advances and perspectives. Frontiers in Microbiology.

[219] Zelante T, Iannitti RG, Cunha C, De Luca A, Giovannini G, Pieraccini G (2013). Tryptophan catabolites from microbiota engage aryl hydrocarbon receptor and balance mucosal reactivity via interleukin-22. Immunity.

[220] Venkatesh M, Mukherjee S, Wang H, Li H, Sun K, Benechet AP (2014). Symbiotic bacterial metabolites regulate gastrointestinal barrier function via the xenobiotic sensor PXR and Toll-like receptor 4. Immunity.

[221] Lamas B, Richard ML, Leducq V, Pham HP, Michel ML, Da Costa G (2016). CARD9 impacts colitis by altering gut microbiota metabolism of tryptophan into aryl hydrocarbon receptor ligands. Nature Medicine.

[222] Kim CH (2018). Immune regulation by microbiome metabolites. Immunology.

[223] Gao J, Xu K, Liu H, Liu G, Bai M, Peng C (2018). Impact of the gut microbiota on intestinal immunity mediated by tryptophan metabolism. Frontiers in Cellular and Infection Microbiology.

[224] Sonner JK, Keil M, Falk-Paulsen M, Mishra N, Rehman A, Kramer M (2019). Dietary tryptophan links encephalogenicity of autoreactive T cells with gut microbial ecology. Nature Communications.

[225] Shi LZ, Faith NG, Nakayama Y, Suresh M, Steinberg H, Czuprynski CJ (2007). The aryl hydrocarbon receptor is required for optimal resistance to Listeria monocytogenes infection in mice. Journal of immunology (Baltimore, Md.: 1950).

[226] Qiu J, Heller JJ, Guo X, Chen Z-ME, Fish K, Fu Y-X (2012). The aryl hydrocarbon receptor regulates gut immunity through modulation of innate lymphoid cells. Immunity.

[227] Zhang L, Nichols RG, Patterson AD (2017). The aryl hydrocarbon receptor as a moderator of host-microbiota communication. Current Opinion in Toxicology.

[228] Auslander N, Yizhak K, Weinstock A, Budhu A, Tang W, Wang XW (2016). A joint analysis of transcriptomic and metabolomic data uncovers enhanced enzyme-metabolite coupling in breast cancer. Science and Reports.

[229] Miller-Fleming L, Olin-Sandoval V, Campbell K, Ralser M (2015). Remaining mysteries of molecular biology: The role of polyamines in the cell. Journal of Molecular Biology.

[230] Seiler N, Bolkenius FN, Rennert OM (1981). Interconversion, catabolism and elimination of the polyamines. Medical Biology.

[231] Goodwin AC, Destefano Shields CE, Wu S, Huso DL, Wu X, Murray-Stewart TR (2011). Polyamine catabolism contributes to enterotoxigenic Bacteroides fragilis-induced colon tumorigenesis. Proceedings of the National Academy of Sciences of the United States of America.

[232] Michael AJ (2018). Polyamine function in archaea and bacteria. The Journal of biological chemistry.

[233] Sittipo P, Shim JW, Lee YK (2019). Microbial metabolites determine host health and the status of some diseases. International Journal of Molecular Sciences.

[234] Loser C, Folsch UR, Paprotny C, Creutzfeldt W (1990). Polyamine concentrations in pancreatic tissue, serum, and urine of patients with pancreatic cancer. Pancreas.

[235] Loser C, Folsch UR, Paprotny C, Creutzfeldt W (1990). Polyamines in colorectal cancer. Evaluation of polyamine concentrations in the colon tissue, serum, and urine of 50 patients with colorectal cancer. Cancer.

[236] Morrison DJ, Preston T (2016). Formation of short chain fatty acids by the gut microbiota and their impact on human metabolism. Gut Microbes.

[237] Clausen MR, Mortensen PB, Bendtsen F (1991). Serum levels of short-chain fatty acids in cirrhosis and hepatic coma. Hepatology.

[238] Jakobsdottir G, Bjerregaard JH, Skovbjerg H, Nyman M (2013). Fasting serum concentration of short-chain fatty acids in subjects with microscopic colitis and celiac disease: No difference compared with controls, but between genders. Scandinavian Journal of Gastroenterology.

[239] Ktsoyan ZA, Mkrtchyan MS, Zakharyan MK, Mnatsakanyan AA, Arakelova KA, Gevorgyan ZU (2016). Systemic concentrations of short chain fatty acids are elevated in Salmonellosis and Exacerbation of Familial Mediterranean Fever. Frontiers in Microbiology.

[240] Pryde SE, Duncan SH, Hold GL, Stewart CS, Flint HJ (2002). The microbiology of butyrate formation in the human colon. FEMS Microbiology Letters.

[241] Thirunavukkarasan M, Wang C, Rao A, Hind T, Teo YR, Siddiquee AA (2017). Short-chain fatty acid receptors inhibit invasive phenotypes in breast cancer cells. PLoS One.

[242] Huang CK, Chang PH, Kuo WH, Chen CL, Jeng YM, Chang KJ (2017). Adipocytes promote malignant growth of breast tumours with monocarboxylate transporter 2 expression via beta-hydroxybutyrate. Nature Communications.

[243] Phelan JP, Reen FJ, Dunphy N, O'Connor R, O'Gara F (2016). Bile acids destabilise HIF-1alpha and promote anti-tumour phenotypes in cancer cell models. BMC Cancer.

[244] Goldberg AA, Titorenko VI, Beach A, Sanderson JT (2013). Bile acids induce apoptosis selectively in androgen-dependent and -independent prostate cancer cells. PeerJ.

[245] Gafar AA, Draz HM, Goldberg AA, Bashandy MA, Bakry S, Khalifa MA (2016). Lithocholic acid induces endoplasmic reticulum stress, autophagy and mitochondrial dysfunction in human prostate cancer cells. PeerJ.

[246] Goldberg AA, Beach A, Davies GF, Harkness TA, Leblanc A, Titorenko VI (2011). Lithocholic bile acid selectively kills neuroblastoma cells, while sparing normal neuronal cells. Oncotarget.

[247] Swales KE, Korbonits M, Carpenter R, Walsh DT, Warner TD, Bishop-Bailey D (2006). The farnesoid X receptor is expressed in breast cancer and regulates apoptosis and aromatase expression. Cancer Research.

[248] Yager JD (2015). Mechanisms of estrogen carcinogenesis: The role of E2/E1-quinone metabolites suggests new approaches to preventive intervention–a review. Steroids.

[249] Vergara, D., Simeone, P., Damato, M., Maffia, M., Lanuti, P., & Trerotola, M. (2019). The cancer microbiota: EMT and inflammation as shared molecular mechanisms associated with plasticity and progression. *Journal of Oncology, 2019*10.1155/2019/1253727 10.1155/2019/1253727 PMC6854237 31772577

[250] Chiba A, Bawaneh A, Velazquez C, Clear KYJ, Wilson AS, Howard-McNatt M (2019). Neoadjuvant chemotherapy shifts breast tumor microbiota populations to regulate drug responsiveness and the development of metastasis. Molecular Cancer Research.

[251] Ingman WV (2019). The gut microbiome: A new player in breast cancer metastasis. Cancer Research.

[252] Menzies KJ, Zhang H, Katsyuba E, Auwerx J (2016). Protein acetylation in metabolism - metabolites and cofactors. Nature Reviews. Endocrinology.

[253] Fellows R, Varga-Weisz P (2020). Chromatin dynamics and histone modifications in intestinal microbiota-host crosstalk. Molecular Metabolism.

[254] Yu X, Shahir AM, Sha J, Feng Z, Eapen B, Nithianantham S (2014). Short chain fatty acids from periodontal pathogens suppress HDACs, EZH2, and SUV39H1 to promote Kaposi's sarcoma-associated herpesvirus replication. Journal of Virology.

[255] Haase S, Haghikia A, Wilck N, Muller DN, Linker RA (2018). Impacts of microbiome metabolites on immune regulation and autoimmunity. Immunology.

[256] Ratajczak W, Ryl A, Mizerski A, Walczakiewicz K, Sipak O, Laszczynska M (2019). Immunomodulatory potential of gut microbiome-derived short-chain fatty acids (SCFAs). Acta Biochimica Polonica.

[257] Fachi JL, Secca C, Rodrigues PB, Mato FCP, Di Luccia B, Felipe JS (2020). Acetate coordinates neutrophil and ILC3 responses against C. difficile through FFAR2. Journal of Experimental Medicine.

[258] Győrffy B (2021). Survival analysis across the entire transcriptome identifies biomarkers with the highest prognostic power in breast cancer. Computational and Structural Biotechnology Journal.

[259] Routy B, Le Chatelier E, Derosa L, Duong CPM, Alou MT, Daillere R (2018). Gut microbiome influences efficacy of PD-1-based immunotherapy against epithelial tumors. Science.

[260] Viaud S, Saccheri F, Mignot G, Yamazaki T, Daillere R, Hannani D (2013). The intestinal microbiota modulates the anticancer immune effects of cyclophosphamide. Science.

[261] Xie W, Huang Y, Xie W, Guo A, Wu W (2010). Bacteria peptidoglycan promoted breast cancer cell invasiveness and adhesiveness by targeting toll-like receptor 2 in the cancer cells. PLoS One.

[262] Lee KH, Kim EY, Yun JS, Park YL, Do SI, Chae SW (2018). The prognostic and predictive value of tumor-infiltrating lymphocytes and hematologic parameters in patients with breast cancer. BMC Cancer.

[263] Ethier JL, Desautels D, Templeton A, Shah PS, Amir E (2017). Prognostic role of neutrophil-to-lymphocyte ratio in breast cancer: A systematic review and meta-analysis. Breast Cancer Research.

[264] Zhang Y-M, Rock CO (2008). Membrane lipid homeostasis in bacteria. Nature Reviews Microbiology.

[265] Van der Merwe M, Van Niekerk G, Botha A, Engelbrecht AM (2021). The onco-immunological implications of Fusobacterium nucleatum in breast cancer. Immunology Letters.

[266] Lakritz JR, Poutahidis T, Mirabal S, Varian BJ, Levkovich T, Ibrahim YM (2015). Gut bacteria require neutrophils to promote mammary tumorigenesis. Oncotarget.

[267] Luu M, Weigand K, Wedi F, Breidenbend C, Leister H, Pautz S (2018). Regulation of the effector function of CD8(+) T cells by gut microbiota-derived metabolite butyrate. Science and Reports.

[268] Absil L, Journé F, Larsimont D, Body JJ, Tafforeau L, Nonclercq D (2020). Farnesoid X receptor as marker of osteotropism of breast cancers through its role in the osteomimetism of tumor cells. BMC Cancer.

[269] Mouchemore KA, Anderson RL, Hamilton JA (2018). Neutrophils, G-CSF and their contribution to breast cancer metastasis. FEBS Journal.

[270] Lehouritis P, Cummins J, Stanton M, Murphy CT, McCarthy FO, Reid G (2015). Local bacteria affect the efficacy of chemotherapeutic drugs. Scientific Reports.

[271] Buchta Rosean C, Feng TY, Azar FN, Rutkowski MR (2019). Impact of the microbiome on cancer progression and response to anti-cancer therapies. Advances in Cancer Research.

[272] Zhang X, Yu L, Shi J, Li S, Yang S, Gao W (2021). Antibiotics modulate neoadjuvant therapy efficiency in patients with breast cancer: A pilot analysis. Scientific Reports.

[273] Plaza-Díaz J, Álvarez-Mercado AI, Ruiz-Marín CM, Reina-Pérez I, Pérez-Alonso AJ, Sánchez-Andujar MB (2019). Association of breast and gut microbiota dysbiosis and the risk of breast cancer: A case-control clinical study. BMC Cancer.

[274] Armstrong, G., Martino, C., Rahman, G., Gonzalez, A., Vázquez-Baeza, Y., Mishne, G., et al. (2021). Uniform Manifold Approximation and Projection (UMAP) Reveals composite patterns and resolves visualization artifacts in microbiome data. *mSystems*, e0069121, 10.1128/mSystems.00691-21. 10.1128/mSystems.00691-21 PMC8547469 34609167

[275] Dakubo, G. D. (2019). Body fluid microbiome as cancer biomarkers.In *Cancer Biomarkers in Body Fluids*. Cham: Springer

[276] Yang J, Tan Q, Fu Q, Zhou Y, Hu Y, Tang S (2017). Gastrointestinal microbiome and breast cancer: Correlations, mechanisms and potential clinical implications. Breast Cancer.

[277] Jarman R, Ribeiro-Milograna S, Kalle W (2020). Potential of the microbiome as a biomarker for early diagnosis and prognosis of breast cancer. Journal of breast cancer.

[278] Dieleman S, Aarnoutse R, Ziemons J, Kooreman L, Boleij A, Smidt M (2021). Exploring the potential of breast microbiota as biomarker for breast cancer and therapeutic response. American Journal of Pathology.

[279] Parida S, Sharma D (2019). The power of small changes: Comprehensive analyses of microbial dysbiosis in breast cancer. Biochimica et Biophysica Acta - Reviews on Cancer.

